# MALDI Mass Spectrometry Imaging Highlights Specific Metabolome and Lipidome Profiles in Salivary Gland Tumor Tissues

**DOI:** 10.3390/metabo12060530

**Published:** 2022-06-08

**Authors:** Eduardo Sommella, Emanuela Salviati, Vicky Caponigro, Manuela Grimaldi, Simona Musella, Alessia Bertamino, Luigi Cacace, Remo Palladino, Giuseppe Di Mauro, Federico Marini, Anna Maria D’Ursi, Pietro Campiglia

**Affiliations:** 1 Department of Pharmacy, University of Salerno, Via Giovanni Paolo II, 132, 84084 Fisciano, Italy; esommella@unisa.it (E.S.); esalviati@unisa.it (E.S.); vcaponigro@unisa.it (V.C.); magrimaldi@unisa.it (M.G.); smusella@unisa.it (S.M.); abertamino@unisa.it (A.B.); dursi@unisa.it (A.M.D.); 2 U.O.C Clinical Pathology D.E.A. III Umberto I, Viale S. Francesco D’Assisi, 84014 Nocera Inferiore, Italy; cacace10luigi@gmail.com (L.C.); remo.palladino@tiscali.it (R.P.); dr.giuseppedimauro@gmail.com (G.D.M.); 3 Department of Chemistry, University of Rome “La Sapienza”, Piazzale Aldo Moro 5, 00185 Rome, Italy; federico.marini@uniroma1.it

**Keywords:** mass spectrometry imaging, MALDI-MSI, metabolomics, lipidomics, parotid tumor, spatial segmentation

## Abstract

Salivary gland tumors are relatively uncommon neoplasms that represent less than 5% of head and neck tumors, and about 90% are in the parotid gland. The wide variety of histologies and tumor characteristics makes diagnosis and treatment challenging. In the present study, Matrix-assisted laser desorption/ionization mass spectrometry imaging (MALDI-MSI) was used to discriminate the pathological regions of patient-derived biopsies of parotid neoplasms by metabolomic and lipidomic profiles. Fresh frozen parotid tissues were analyzed by MALDI time-of-flight (TOF) MSI, both in positive and negative ionization modes, and additional MALDI-Fourier-transform ion cyclotron resonance (FT-ICR) MSI was carried out for metabolite annotation. MALDI-TOF-MSI spatial segmentation maps with different molecular signatures were compared with the histologic annotation. To maximize the information related to specific alterations between the pathological and healthy tissues, unsupervised (principal component analysis, PCA) and supervised (partial least squares-discriminant analysis, PLS-DA) multivariate analyses were performed presenting a 95.00% accuracy in cross-validation. Glycerophospholipids significantly increased in tumor tissues, while sphingomyelins and triacylglycerols, key players in the signaling pathway and energy production, were sensibly reduced. In addition, a significant increase of amino acids and nucleotide intermediates, consistent with the bioenergetics request of tumor cells, was observed. These results underline the potential of MALDI-MSI as a complementary diagnostic tool to improve the specificity of diagnosis and monitoring of pharmacological therapies.

## 1. Introduction

Salivary gland disorders are rare diseases that occur at a rate of about 1–3 cases per 100,000 people per year (in different countries) [1,2] and represent around 5% of all head and neck neoplasms [3]. The parotid gland is the most frequent site of salivary gland tumors, although only 25% of such lesions are malignant [4]. Benign parotid neoplasms, although not aggressive, are excised in most cases, since they can continue to grow, compressing adjacent regions and becoming very painful as well as unsightly, and, at worst, turn malignant [5].

Due to the heterogeneous cellular compositions of parotid tissues and the diverse histological appearances of tumor lesions, the recognition of a tumorous mass, as well as the distinctions between tumor types, can be difficult, particularly based on the usual diagnosis tools, such as fine-needle aspiration (FNA) or a core needle biopsy [6]. In addition to the overlapping morphologic patterns of salivary gland tumors, the complex evaluations of cytological parameters in the various histotypes do not follow (in some cases) the canonical morphological correlations, making an accurate differential diagnosis between benign and malignant lesions, particularly impervious. Metabolic reprogramming is a hallmark of cancer and can be exploited for both diagnostic and therapeutic purposes [7,8]. In this regard, mass spectrometry-based metabolomics allows for monitoring the main molecular changes related to cancer cell signaling, disease onset mechanisms, and tumor progression [9], and has increasingly emerged as a valid tool for early diagnosis and to identify predictive biomarkers of cancer [10]. Gas chromatography–mass spectrometry (GC–MS) and nuclear magnetic resonance (NMR) are usually employed for metabolome analyses of clinical samples [11,12], even though high-resolution mass spectrometry (HRMS) in direct infusion (DI) and/or coupled to ultra-high performance liquid chromatography (UHPLC–HRMS) has become the gold standard for metabolomics [13]. However, these approaches require tissue homogenization prior to extraction and analysis and are unable to provide detailed information concerning the spatial localization of key metabolites. For this purpose, mass spectrometry imaging (MSI) offers an unbiased, label-free visualization of the spatial distributions of biomolecules; it is highly useful to correlate the modulations with different histopathological features of the tumor tissue, allowing further insights into the tumor environment [14], and improving or individuating individualized therapies.

It has been demonstrated that lipid metabolism perturbations are associated with many cancer types [15], including oral cavity cancer [16,17]. The complex lipid metabolic changes may be explained by the high proliferation rate of cancerous cells leading to continuous cell membrane synthesis, disruption of energy homeostasis, sustained cell signaling, and protein distribution, with variations in the serum or tissue levels of phospholipid components [18]. In this regard, previous MALDI imaging data have highlighted global lipidomic changes associated with head and neck cancer tissues [17,19]. In particular, phosphatidylcholine distribution (visualized through MSI) has specifically increased in the Warthin tumor lymphoid stroma of salivary glands [20]. The Warthin tumor is the second most common tumor of the parotid gland (after pleomorphic adenoma) with a bilayer of epithelial cells resting on a dense lymphoid stroma. Adenolymphoma, also called papillary cystadenoma lymphomatosum papilliferum, is the only benign neoplasm of salivary glands associated with smoking and arises from the incorporation of lymphoid tissue in the parotid gland and induction of cystic and oncocytic changes by inflammatory infiltration. Besides lipid metabolism, alterations in nucleotide biosynthesis, glycolysis, the tricarboxylic acid (TCA) cycle pathway, and amino acid metabolism represent common traits of tumor cells [21]. Specifically, the metabolome profiles in the saliva of the neck and oral cancer have suggested an alteration, mainly in the metabolic pathways of glycogenic and ketogenic amino acids [16,22]. Despite this, polar metabolite imaging of pathological regions in parotid neoplastic tissues has been poorly investigated. In this regard, the concomitant investigation of multi-omic alterations induced by tumor cells could improve the comprehension of the complex metabolic mechanisms involved in the physiopathology of salivary gland tumors. Hence, in the present study, MALDI-MSI was applied to investigate metabo-lipidomic differences between healthy and tumor regions of patient-derived biopsies of the parotid tissues. Applying spatial segmentation and unsupervised and supervised multivariate approaches for data analyses, characteristic metabo-lipidomic signatures have been assigned to tumor and healthy adjacent parotid sections, underlying the growing energy request and continuous cellular proliferation of neoplastic tissues.

## 2. Results and Discussion

### 2.1. Histopathological Assessment

All corresponding H&E-stained sections were independently annotated by an expert pathologist (L.C.) to distinguish the tumor and non-tumor areas by their histological features (Figure S1).

Microscopic descriptions of Warthin tumor areas exhibited variable proportions of papillary-cystic structures lined with bilayer of epithelial cells, consisting of oncocytic columnar cells with underlying discontinuous basal cells and resting on dense lymphoid stroma with variable germinal centers. Lymphocytes are typically small–mature and have high nucleus–cytoplasm ratios with deeply-stained nuclei and relatively small amounts of cytoplasms. Oncocytes show densely granular cytoplasms (stuffed with mitochondria on electron microscopy), centrally located nuclei, and small nucleoli.

To test the applicability of the approach to different histopathological conditions, pleomorphic adenoma and salivary glands with chronic sialoadenitis were also included in the study. The section of pleomorphic adenoma has epithelial, myoepithelial, and stromal components. The histopathologic appearance shows a tumor with a lobulated growth pattern with tubular and acinar structures formed by the epithelial component mixed with myoepithelial cells in the background of the myxoid stroma. In chronic sialoadenitis, the histopathological area shows various degrees of acinar destruction, fibrosis, and chronic and active periductal inflammation with lymphoid aggregates and focal granulomatous reaction. Ducts may undergo squamous and mucous metaplasia. Microliths can be seen.

The normal parotid salivary gland tissue consists of secretory acini, serous and mucous, and intercalated ducts, which are lined by a simple low cuboidal epithelium and surrounded by myoepithelial cells. The glands are divided into lobules by connective tissue septa and there are adipocytes between acini.

### 2.2. MALDI-MSI of Polar and Non-Polar Metabolites in Parotid Sections

In the current study, MALDI-MSI methods were developed to map both lipids and metabolites in consecutive sections of 22 fresh frozen human parotid tissue samples (*n* = 11 patients). DHB and 9-AA were selected for MALDI-positive and -negative, respectively, MSI analyses, to detect the highest possible number of metabolites.

Metabolome and lipidome profiling by MALDI-TOF MSI were performed at a lateral resolution of 50 µm. For the technical repeatability evaluation of the experiments, three technical replicates of two biological replicates were performed. In this regard, the coefficient of variation (CV%) relative to the average peak area was 15.19% ± 14.49 for the positive ion mode and 6.09% ± 6.72 for negative ionization, indicating satisfying repeatability for both metabolite classes.

The molecules imaged were initially identified using the accurate mass information provided by MALDI-FT-ICR MS, which delivers sub-ppm mass accuracy and high resolution (>100,000) together with isotopic fine structure (ISF) of the FT-ICR measurement. This results in resolving metabolite peaks with similar nominal masses in full-scan mode providing accurate molecular formula measurements [23]. The annotation was also performed “off-tissue” by UHPLC–HRMS/MS [24,25] (Supplementary Materials Section S1).

A total of 354 compounds were annotated, 301 lipids and 53 metabolites (Table S1), which were mainly divided into: amino acids, peptides, and analogues (30%); carbohydrates and carbohydrate conjugates (4%); carboxylic acids and derivatives (6%); purine and pyrimidines derivatives (2%); quinolines and derivatives (0.3%), fatty acyls (FA) (14%); glycerophospholipids (GP) (glycerophosphocholines 31%, glycerophosphoethanolamines 12%, glycerophosphoglycerols 0.7%, glycerophosphoinositols 2%, glycerophosphoserines 8%, and glycerophosphates 6%), sphingolipids (SP) (13%), sterol lipids (ST) (4%), and glycerolipids (GL) (9%).

### 2.3. Exploratory Analysis, Classification, and Model Validation

#### 2.3.1. Principal Component Analysis (PCA)

**Figure 1:**
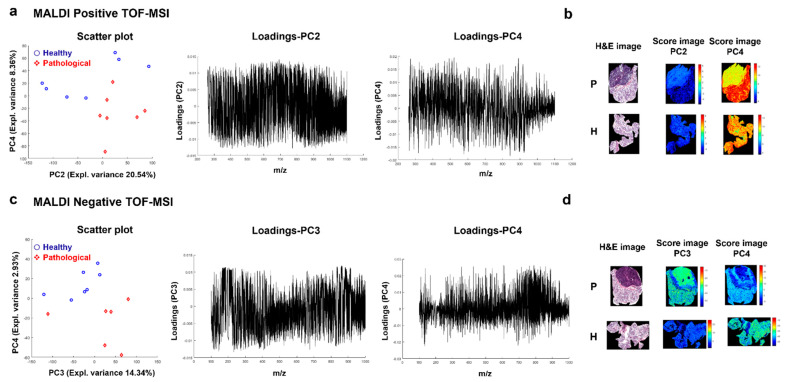
Principal component analysis (PCA) results of MALDI-positive and -negative TOF-MSI data analysis. (**a**) PC2 vs. PC4 scores with loadings plots and (**b**) PC2 and PC4 score images for patient 3 compared with the histological image for the positive ion mode MSI dataset. (**c**) PC3 vs. PC4 scores with loadings plots and (**d**) PC3 and PC4 score images for patient 3 compared with the histological image for the negative ion mode MSI dataset. PCA was performed using pre-processed spectra from the training set. Loadings obtained from PCA were applied to the images of the training set. Abbreviations: H&E, Hematoxylin and Eosin staining; P, pathological tissue; H, healthy tissue.

The principal component analysis (PCA) was carried out on the auto-scaled training set for MALDI, both positive and negative TOF-MSI datasets. A clear difference between the pathological and healthy spectra is evident in the positive ion mode scores plot (Figure 1a). The PC2 (explained variance 20.54%) and PC4 (explained variance 8.36%) spaces separated almost perfectly the two types of tissues. The interpretation of the value of the loadings is complicated due to the high density of the information.

The obtained loadings were applied to the images of both training and test sets in order to build score maps. The score maps obtained by the application of PC2 and PC4 loadings to the training set presented a clear difference between the pathological and healthy sets (Figure S2). However, patient 4 and patient 5 tissues did not seem to follow the general trend. The discrepancies may be due to the different tumor histology of patient 5 (pleomorphic adenoma). The scores patterns found on the test set are comparable with those of the training set (Figure S3). The PC2 and PC4 score maps for patient 3 are reported as an example in Figure 1b.

A clear difference between the pathological and healthy tissues is present for the negative ion mode spectra and reported in the object-level score plot (Figure 1c). The spaces of PC3 (explained variance 13.94%) and PC4 (explained variance 2.93%) show the two clusters using the median spectra. The score maps resulting from the application of loadings obtained by PCA are reported in Figures S4 and S5 for the training and test sets, respectively. The training score images show intensity differences between healthy and pathological tissues. This difference is less strong for the test set. However, this trend may reflect the different tumor histological types of patients 2 and 11, part of the test set, diagnosed as chronic sialoadenitis. The score maps of both pathological and healthy tissues for patient 3, showing a strong difference between the two types, are reported in Figure 1d.

#### 2.3.2. Partial Least Squares-Discriminant Analysis (PLS-DA)

The partial least squares-discriminant analysis (PLS-DA) model was built using the median spectra of the entire image for the training set in order to discriminate between pathological and healthy tissues. Then, the optimal model was applied to the images of both the training and test sets. The optimal number of latent variables (nLVs), and so the optimal model, was selected in order to maximize the accuracy and minimize the classification error in cross-validation. Both images, pathological and healthy, collected on a specific patient were left out at each iteration.

**Figure 2:**
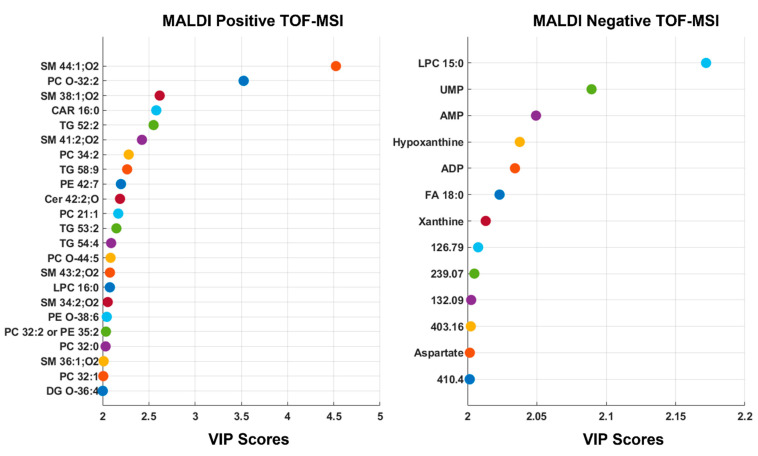
Partial least squares-discriminant analysis (PLS-DA) of MALDI-positive and -negative TOF-MSI data. Graphical illustration of the most relevant predictors for the models built on positive (**left**) and negative (**right**) ion mode MSI analyses based on their values of the variable importance in projection (VIP) index, using as threshold a VIP score > 2.

Variable importance in projection (VIP) indices were calculated for each model. The variables with VIP scores > 2 are displayed in Figure 2 for both positive and negative modes. Then, a new model was calculated using only the VIP-based selected variables. The PLS-DA performances for both positive and negative ion modes, respectively, for training, cross-validation, and the test set, are reported in Table 1. Sensitivity and specificity are defined with respect to the healthy class. The optimal model was found at 3 LVs for the positive ion mode analysis, resulting in 100% accuracy, sensitivity, and specificity in cross-validation. Selecting the variables based on the VIP scores—so reducing the number of predictors to 441—the accuracy, sensitivity, and specificity were 100% in cross-validation for 1 LV. For the negative ion mode, the optimal model for the entire range was found using 3 LVs, leading to accuracy, sensitivity, and specificity of 92.68%, 100.00%, and 85.71%, respectively, in cross-validation. Reducing the variables to 245, the performances of the optimal model, built using 3 LVs, improved to 100% for accuracy, sensitivity, and specificity. Accuracy, sensitivity, and specificity for the test set were calculated over the entire image considering the classification of each pixel. The heterogeneous nature of the pathological tissues makes these values less reliable per se. So, it is important to consider the classifications maps reported in Figures S6–S9 for the positive ion mode analysis and in Figures S10–S13 for the negative ion mode analysis. The percentage of correctly classified pixels is reported for each image. For the positive ion count analysis, comparing the classification maps with the staining images, it is evident that reducing the number of variables improves the localization of the pathological pixels (Figures S8 and S9). All tissues diagnosed as the Warthin tumor were correctly classified for both training and test sets. However, some pixels were misclassified for the healthy tissue of patient 2, but the classification improved, reducing the number of variables. As previously mentioned, this may be connected to the different tumor histologies of the tissues. In addition, the pathological tissue for patient 11 presented correctly classified pixels localized in specific regions.

For the negative ion mode analysis, the number of misclassified pixels increased reducing the number of variables. However, all images of the training set were correctly classified when all predictors were used. By observing the maps for the test set (Figure S11), it is evident that the model cannot identify the pathology of patient 11.

### 2.4. Spatial Segmentation of MALDI-MSI Data and Specific m/z Values Colocalize with Healthy or Tumor Parotid Region

**Figure 3:**
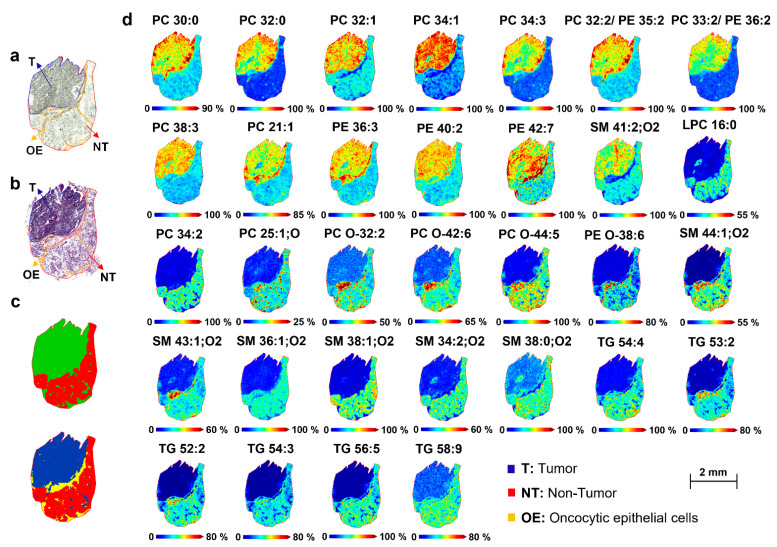
Molecular distribution of lipid species between the tumor and healthy regions of patient-derived parotid sections. (**a**) Optical image and (**b**) Hematoxylin and Eosin (H&E) image of parotid tissue with the pathologist’s annotation of the regions. (**c**) Bisecting the K-means segmentation map generated from the MALDI-positive TOF-MSI dataset. (**d**) MALDI-MS images of relevant lipids measured in positive ion mode, comparing the distribution between tumor and healthy regions of the parotid tissue. [PC 30:0+K]^+^, *m/z* 744.49; [PC 32:0+K]^+^, *m*/*z* 772.59; [PC 32:1+K]^+^, *m/z* 770.55; [PC 34:1+K]^+^, *m/z* 798.54; [PC 34:3+H]^+^, *m/z* 756.56; [PC 32:2 or PE 35:2+H]^+^, *m/z* 730.53; [PC 33:2 or PE 36.2+H]^+^, *m/z* 744.57; [PC 38:3+H]^+^, *m/z* 812.62; [PC 21:1+H]^+^, *m/z* 578.41; [PE 36:3+H]^+^, *m/z* 742.53; [PE 40:2+H]^+^, *m/z* 800.55; [PE 42:7+H]^+^, *m/z* 818.47; [SM 41:2;O2+Na]^+^, *m/z* 821.66; [LPC 16:0+H]^+^, *m/z* 496.43; [PC 34:2+K]^+^ *m/z* 758.57; [PC 25:1;O+H]^+^, *m/z* 650.46; [PC O-32:2+H]^+^, *m/z* 551.51; [PC O-42:6+Na]^+^, *m/z* 870.65; [PC O-44:5+Na]^+^, *m/z* 900.74; [PE O-38:6+H]^+^, *m/z* 750.54; [SM 44:1;O2+K]^+^, *m/z* 881.66; [SM 43:2;O2+H]^+^, *m/z* 827.68; [SM 36:1;O2+H]^+^, *m/z* 731.60; [SM 38:1;O2+Na]^+^, *m/z* 781.59; [SM 34:2;O2+Na]^+^, *m/z* 723.57; [SM 38:0;O2+H]^+^, *m/z* 761.60; [TG 54:4+H]^+^, *m/z* 883.77; [TG 53:2+Na]^+^, *m/z* 895.77; [TG 52:2+Na]^+^, *m/z* 881.76; [TG 54:3+Na]^+^, *m/z* 907.79; [TG 56:5+H]^+^, *m/z* 909.79; [TG 58:9+H]^+^, *m/z* 929.76. Detailed parameters for tentative annotation are reported in Table 2 and Table S2. Scale bar = 2 mm. Color scale bars are shown as percentages of the maximum intensity and were adjusted for each ion image to show a clear distribution. Data were normalized to the total ion count (TIC) of each individual spectrum. Lateral resolution, 50 μm. Abbreviations: T, tumor region; NT, non-tumor region; OE: oncocytic epithelial cells.

**Figure 4:**
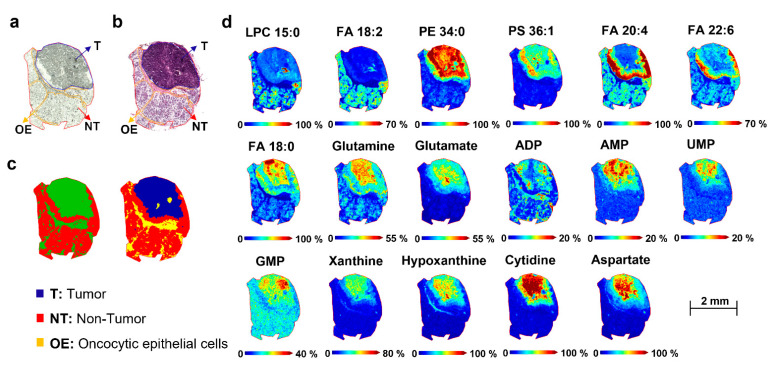
Molecular distribution of non-polar and polar metabolites between tumor and healthy regions of patient-derived parotid sections. (**a**) Optical image and (**b**) Hematoxylin and Eosin (H&E) image of parotid tissue with the pathologist’s annotation of the regions. (**c**) Bisecting the K-means segmentation map generated from the MALDI-negative TOF-MSI dataset. (**d**) MALDI-MS images of relevant lipids and metabolites measured in negative ion mode, comparing the distribution between tumor and healthy regions of the parotid tissue. [LPC 15:0-H]^−^, *m/z* 480.32; [FA 18:2-H]^−^, *m/z* 279.24; [PE 34:0-H]^−^, *m/z* 718.54; [PE 34:0-H]^−^, *m/z* 718.54; [PS 36:1-H]^−^, *m/z* 524.33; [PS 36:1-H]^−^, *m/z* 524.33; [FA 20:4-H]^−^, *m/z* 303.22; [FA 22:6-H]^−^, *m/z* 327.20; [FA 18:0-H]^−^, *m/z* 283.27; [Glutamine-H]^−^, *m/z* 145.07; [Glutamate-H]^−^, *m/z* 146.05; [ADP-H]^−^, *m/z* 426.04; [AMP-H]^−^, *m/z* 346.06; [UMP-H]^−^, *m/z* 323.06; [GMP-H]^−^, *m/z* 362.06; [Xanthine-H]^−^, *m/z* 151.02; [Hypoxanthine-H]^−^, *m/z* 135.04; [Cytidine-H]^−^, *m/z* 242.06; [Aspartate-H]^−^, *m/z* 132.04. Detailed parameters for the tentative annotation are reported in Table 2, Tables S2 and S3. Scale bar = 2 mm. Color scale bars are shown as percentages of maximum intensity and were adjusted for each ion image to show a clear distribution. Data were normalized to the total ion count (TIC) of each individual spectrum. Lateral resolution, 50 μm. Abbreviations: T, tumor region; NT, non-tumor region; OE: oncocytic epithelial cells.

To further obtain unsupervised clustering of the non-tumor and tumor regions, the intra-tissue heterogeneity was visualized by the spatial segmentation approach. The segmentation map represents the spatial molecular information of a sample without any prior histological knowledge [26] and displays a molecular profile (different *m/z* values corresponding to molecules) associated with each cluster [27]. MALDI-TOF MSI datasets were used for the spatial segmentation analysis. The spatial segmentation algorithm was applied to each tissue. All pixels with similar spectra were grouped into two and four colored clusters. All pixels corresponding to the same cluster were represented by the same color. The spatial segmentation was consistent with the histopathological stain and annotation, which enabled distinguishing the tumor from healthy adjacent parotid regions, as well as the epithelial or connective tissues, as reported in Figure 3a–c and Figure 4a–c, for patient 3, as representative tissue.

Subsequently, colocalized *m/z* values were correlated to the specific ROI of the segmentation map by applying Pearson’s correlation. Based on this approach, only *m/z* values with high correlation coefficients (≥0.5) were selected (Table 2).

The segmentation pipeline was able to confirm nine *m/z* values that discriminated between tumor and healthy groups in PLS-DA and revealed additional molecular distinctions within the tumor and non-tumor regions. In the positive ion mode, the selected ion images *m/z* 742.53, 744.49, 744.58, 756.56, 772.59, 798.54, 800.55, 812.62, and 870.65 were correlated to the tumor tissue, while *m/z* 650.46, 712.46, 723.56, 731.60, 750.53, 758.57, and 781.58 were localized in the non-tumor adjacent region. Regarding the MALDI-negative MSI analysis, *m/z* 132.03, 135.04, 145.06, 146.04, 242.06, 346.06, 362.05, 524.33, and 718.54 were distributed in the pathological region, and *m/z* 279.24, 303.22, and 327.20 in the healthy region (Figure 3d and Figure 4d).

### 2.5. Spatial Discrimination of Metabolome and Lipidome Profiles in Parotid Gland Tumor Tissues by MALDI-MSI

This study aimed to highlight spatial-omics signatures able to discriminate between the non-tumor and tumor regions of parotid biopsies by integrating MALDI-MSI and two computational evaluation strategies, PLS-DA and spatial segmentation. Table 2 reports the putative annotation of lipids and metabolites with a VIP score > 2 and/or Pearson’s correlation coefficients ≥ 0.5, which were most influential for the separation between tumor and healthy tissues. Detailed information for annotation is reported in Tables S2 and S3. The representative images of peaks (*m*/*z*) with significantly different signal intensities in the tumor and non-tumor areas are shown in Figure 3d and Figure 4d.

#### 2.5.1. Lipidome Differences between Tumor and Non-Tumor Areas of Parotid Biopsies

The radical modification of lipid metabolism is one of the most distinctive features of cancer and plays a fundamental role in cell growth, cell signaling, and survival in changing environments [28]. Glycerophospholipids, such as phosphatidylcholines (PCs) and phosphatidylethanolamine (PEs), were among the main contributors to region separation. PC 21:1, PC 30:0, PC 32:0, PC 32:1, PC 32:2 or PE 35:2, PC 33:2, PC 34:1, PC 34:3, PC 38:3, and the ether-linked PC O-42:6 increased in the tumor microenvironment compared with the adjacent normal tissue. These findings are in good agreement with previous studies that have proposed the spatial confinement of PCs to specific cancer models [29,30,31]. It is well known that PCs are the most predominant components of phospholipids for biological membranes; PC metabolism is altered in the onset and development of many tumors [32,33]. In particular, the elevated levels of PC 32:1 and PC 34:1 have been positively correlated to the cancerous areas of colorectal, prostate, and breast cancer tissues [29,30,31]. Interestingly, PC 32:0 was previously found discriminatory in the Warthin tumor region of benign salivary gland tumor tissues analyzed by MALDI-MSI [20]. In addition, PE 34:0, PE 36:2, PE 36:3, PE 40:2, and PE 42:7 have presented increased expressions in the parotid tumor tissues, consistent with the PE behavior observed in other cancer types, including the trend of PE 36:2 observed in breast tumor xenografts analyzed by MALDI-MSI [34].

Conversely, the distribution patterns of PC 34:2, PC O-38:4, and PE O-38:6 were reduced in the parotid tumor regions, in accordance with a previous MALDI-MSI lipidomic analysis in the same cancerous model of two patient-derived biopsies [19].

Moreover, the levels of Lysophosphatidylcholines (LPC) 16:0 decreased in the tumor in comparison to the adjacent healthy tissue. This result is consistent with previous relevant findings in which LPC 16:0 significantly decreased in laryngeal cancer serum samples and prostate cancer tissues, highlighting the early diagnostic potential of this lipid biomarker [33,35]. This point can be supported by the mutual conversion of PCs and LPCs. In fact, upregulating the PC level, which may come from LPC conversion, the LPC level for the tumor decreases [33].

Interestingly, the dysregulations of several sphingomyelins (SMs) and triacylglycerols (TGs) were observed, which have never (or poorly) been investigated for other forms of oral and neck tumors [17,19,36].

All SMs statistically significant (SM 44:1;O2, SM 43:2;O2, SM 36:1;O2, SM 38:1;O2, SM 34:2;O2, SM 38:0;O2) except SM 41:2;O2, were downregulated in the parotid tumor regions. Sphingolipids are essential bioactive components of the cell membrane, and their metabolisms, produce bioactive signaling molecules that modulate fundamental functions within the signal transduction networks in cancer cells [37]. In this regard, the decrease in the levels of SMs in cancer tissues may be associated with the activation of sphingomyelinase by the metabolic products of arachidonic acid, as previously stated for prostate and bladder cancers [38,39,40]. However, in other forms of cancer, such as breast and colorectal cancers, the levels of SM in the tumor region were significantly higher than in peritumor tissue and normal tissue, due to the reduced activity of the alkaline sphingomyelinase, which converts SM to ceramide [41].

Remarkably, a higher level of arachidonic acid (FA 20:4), together with two other fatty acids, FA 18:2 and FA 22:6, were observed in the non-tumor region compared with adjacent cancerous parotid tissue.

Compared to normal tissue, parotid cancer tissues had a lower content of TGs, in particular TG 54:4, TG 53:2, TG 52:2, TG 54:3, TG 56:5, and TG 58:9. These findings are in line with the rapid degradation of TG for energy production and enhanced synthesis of membrane lipids, necessary for the rapid proliferation of cancer cells [41].

#### 2.5.2. Metabolome Differences between Tumor and Non-Tumor Areas of Parotid Biopsies

As cancer is known to affect metabolic pathways and intermediate products, effective monitoring of these changes can provide vital clues in cancer diagnostics. Salivary metabolite profiling by the LC–MS and NMR platforms is emerging to diagnose or screen oral cancer [16,22]. However, to the best of our knowledge, no prior study has evaluated spatial metabolome modulation directly in relation to parotid neoplastic tissue sites. In the present study, the comparison between tumor and healthy regions of the parotid tissues confirmed the differential regulation of metabolic pathways involved in the biosynthetic substrate production for increased cell proliferation; most of these metabolites were consistent with previously reported salivary biomarkers. Among the amino acids, glutamine *m/z* 145.06 and glutamate *m/z* 146.04, previously associated with oral cavity carcinoma [42,43], significantly increased in the parotid tumor region. This is consistent with the recognized importance of glutamine in the bioenergetics request of cancerous cells [9]. Moreover, upon its conversion to glutamate, glutamine provides a key source of carbon for the TCA cycle [44]. An increased level of aspartate *m/z* 132.03 suggested that the glutamate metabolism was also affected in tumor tissue [16]. Among purine metabolism, a change in the abundance of hypoxanthine *m/z* 135.03 and xanthine *m/z* 151.021 in cancerous tissue was confirmed [43]. Hypoxanthine is an upstream metabolite in the nucleotide biosynthetic pathway and, together with the upregulation of cytidine m/z 242.08, adenosine monophosphate (AMP) *m/z* 346.06, adenosine diphosphate (ADP) *m/z* 426.043, guanosine monophosphate (GMP) *m/z* 362.05, uridine monophosphate (UMP) m/z 323.05, indicate a possible increase in the nucleotide metabolism [45]. Overall, the metabolomic profile carried out by MALDI-MSI points out a significant modulation of key markers of parotid cancer and crosstalk with multiple molecular pathways.

## 3. Materials and Methods

### 3.1. Participants and Sample Collection

The study population consisted of 11 patients subjected to surgical resection between November 2020 and January 2022 in the Department of Otolaryngology, DEA III Liv. Nocera-Pagani, Salerno (SA), Italy, for parotid tumors. According to the diagnostic protocols, participants had been previously diagnosed with Warthin tumor (7 subjects), pleomorphic adenoma (1 subject), and salivary gland sites from chronic sialoadenitis (3 subjects). None of the patients included in the study received pharmacological therapies or radiotherapy prior to surgery. Immediately after surgical resection, the 22 biopsies (*n* = 11 for tumor and *n* = 11 for healthy tissues) were quickly snap-frozen and stored at −80 °C. Demographic characteristics and clinical data of the patients are reported in Table 3:

### 3.2. Chemicals

Unless otherwise described, all solvents and additives were LCMS grade and purchased by Merck (Darmstadt, Germany).

### 3.3. Tissue Sample Preparation for MALDI-MSI

The parotid samples were sectioned using a cryostat microtome (Leica CM1950, Leica Microsystems, Wetzlar, Germany) at a thickness of 12 μm, at −20 °C. Tissue sections were thaw-mounted onto indium tin oxide (ITO)-coated glass slides (Bruker Daltonics, Bremen, Germany) and stored at −80 °C until analysis.

### 3.4. Sample Preparation for MALDI-MSI Analysis of Lipids and Metabolites

ITO slides with mounted parotid sections were removed from −80 °C storage and were immediately dried in a vacuum desiccator for 1 h before the MALDI matrix application. Optical images were acquired before the matrix application using a reflecta^®^ MF5000 scanner (reflecta^®^ gmbh, Eutingen im Gäu, Germany) with HistoView Tissue Scanner II software v1.00.90. Moreover, 2,5-dihydroxybenzoic acid (DHB) was used as the matrix for the positive ionization mode MALDI-MS imaging at a concentration of 15 mg/mL in a solution of 0.1% TFA in ACN/H_2_O 90:10 (*v*/*v*). It was applied using an automated sprayer (TM-Sprayer, HTX Technologies, Chapel Hill, NC, USA). The nozzle temperature was set at 60 °C, with a nitrogen gas pressure of 10 psi, a flow rate of 125 μL/min, a nozzle velocity of 1200 mm/min with fourteen passes, 2 mm of track spacing, and a crisscross (CC) spray pattern.

For the negative-mode MALDI-MSI analysis, the 9-aminoacridine (9-AA) matrix was dissolved in 70% EtOH in H_2_O (concentration 10 mg/mL). An automated pneumatic sprayer (TM-Sprayer, HTX Technologies, Chapel Hill, NC, USA) was used to spray the solution over the tissue sections in four passes at 90 °C, with a nitrogen gas pressure of 6 psi, a flow rate of 120 μL/min, a nozzle velocity of 1200 mm/min, 2 mm of track spacing, and a CC spray pattern.

### 3.5. MALDI-MSI Analysis

All MALDI-MSI experiments were performed on a rapifleX^®^ Tissuetyper MALDI mass spectrometer (Bruker Daltonics, Bremen, Germany) equipped with a Smartbeam 3D laser (under “Single”) and with a digitizer frequency of 1.25 GHz. The analyzer was operated in the reflector mode, and the laser was fired with a repetition rate of 10 kHz. Lipidome measurements were performed in a positive mode across the *m*/*z* range of 300–1000, while the data for both lipids and metabolites in the negative ionization mode were acquired in the *m*/*z* range of 100–850. A total of 200 laser shots were fired at each sampling position and the laser power was optimized at the start of each run and then held constant during the experiments. The external calibration was performed using red phosphorous dissolved in 50% acetone, spotted beside the tissue section [46]. All analyses were performed at a lateral resolution of 50 μm.

### 3.6. Metabolite and Lipid Annotation by MALDI-FT-ICR-MS

Additional MALDI imaging experiments were performed to aid in lipid and metabolite annotation [34,47] using a SolariX XR 7T-ESI/MALDI-Fourier-transform ion cyclotron resonance mass spectrometer (Bruker Daltonics, Bremen, Germany) equipped with a Smartbeam II 2 kHz laser. All imaging experiments were performed at a lateral resolution of 100 µm, using a total of 100 laser shots per pixel with a small laser spot size. Data were collected in positive ionization mode across the *m/z* range of 300–1000 using 2 million data points, while negative MS spectra were collected over the *m/z* range of 100–850 using 1 million data points. Ion transmission voltage parameters were set as follows—funnel RF amplitude 120.0 Vpp, RF amplitude TOF 350.0 Vpp, TOF 0.6 ms, and RF frequency transfer optic 4 MHz. All the methods were externally calibrated through the electrospray ion source and NaTFA clusters. Internal calibration was applied using different lock masses—the [PC (16:0/18:1) + H]^+^ (*m/z* 760.58508), the [PC (16:0/18:1) + Na]^+^ (*m/z* 782.56702), and [PC (16:0/18:1) + K]^+^ (*m/z* 798.54096) were used for the MALDI-positive FT-ICR MSI analysis, and the 9-AA cluster ion (*m/z* 193.0771) for the MALDI-negative FT-ICR MSI experiments.

### 3.7. Imaging Data Analysis

Tissue sections were analyzed in a random order to prevent any possible bias due to matrix degradation or variations in mass spectrometer sensitivity.

All MSI data were first visualized using flexImaging (Version 6.0, Bruker Daltonics, Bremen, Germany), and then reduced spectra were imported into SCiLS Lab software (Version 2021a Pro, GmbH, Bremen, Germany) for baseline correction, normalization, peak picking, and peak lists visualization. Measurements with the same matrix and ion mode were merged into one dataset in SCiLS Lab.

For baseline removal, the top hat algorithm convolution was applied with sigma 20; TOF spectra were normalized to the total ion count (TIC) of each individual spectrum, while FT-ICR spectra were normalized against the root mean square (RMS) of all data points. A weak denoising deterministic installation for both the polarities was applied.

A MALDI-FT-ICR MSI data analysis was performed with MetaboScape 2021 (Bruker Daltonics, Bremen, Germany). The mean spectra of each region of interest (ROI) (both tumor and healthy sections) were exported from SCiLS Lab and opened in the MetaboScape 2021. The bucket table was created using the T-ReX^2^ (MALDI Imaging) algorithm. A total of 200 speckles per ROI were created by averaging the spectra within an area of the size of the width-by-height (4:3 ratio), resulting in 85% covered pixels. The spectra were processed in a positive mode using H^+^ as the primary ion, Na^+^ and K^+^ as potential adducts, while in the negative mode, H^−^ was set as the primary ion and Cl^−^ as a potential adduct. For the metabolite annotation, the assignment of the molecular formula was performed for the detected features using Smart Formula™ (SF). The bucket table was annotated with a list of metabolites and lipids obtained, respectively, from the HMDB (https://hmdb.ca/ (accessed on 5 June 2022)) and LIPIDMAPS databases (www.lipidmaps.org (accessed on 5 June 2022)). Annotation was performed with 0.2 ppm (narrow) or 5 ppm (wide) mass tolerance and a mSigma value below 250; molecular formulae were manually inspected considering the most probable adduct form.

### 3.8. Liquid Chromatography–Mass Spectrometry Analysis

Additional UHPLC–HRMS/MS methods were employed to enforce MALDI-MS imaging results. Contiguous tissue slices were collected, lyophilized, and extracted [48]. Hydrophilic interaction liquid chromatography (HILIC)-HRMS was employed for polar metabolite samples, while lipid analyses were performed by reversed-phase (RP)-trapped ion mobility mass spectrometry (TIMS), as previously optimized [49]. Detailed information about the sample preparation, LC–HRMS conditions, and data processing are reported in Supplementary Material Section S1.

### 3.9. Hematoxylin and Eosin (H&E) Stains to MSI Co-Registration

Following MSI analysis, the MALDI matrix was removed by submerging the slide in 95% ethanol for 30 s, then the sections were fixed in two successive 5 min washes in 100% ethanol and were stained by H&E. The slides were cover-slipped and scanned using a reflecta^®^ MF5000 scanner (reflecta^®^ gmbh, Eutingen im Gäu, Germany). The whole slide H&E images were imported to SCiLS and overlaid with the MSI data.

### 3.10. Statistical Analysis by SCiLS Lab Software

The standard segmentation pipeline of the SCiLS Lab software (Version 2021a Pro, GmbH, Bremen, Germany) was applied for automatic peak picking, with a binning of 0.2 bandwidth [50]. The spatial segmentation algorithm (Bisecting k-means) was applied to each tissue to display intratissue heterogeneity. For each resulting cluster, spatial *m/z* colocalized values were searched by calculating Pearson’s correlations between the spatial masks of the cluster and each *m/z* value and taking the *m/z* values with the highest correlation values (*r* > 0.5).

### 3.11. Multivariate Data Analysis

#### 3.11.1. Data Pre-Processing

MALDI imaging data files normalized to TIC were converted into the imzML format using SCiLS Lab software (Version 2021a Pro, GmbH, Bremen, Germany) and imported in Matlab R2021a (The MathWorks Inc, Natick, MA, USA) using a modified version of the imzMLConverter [51]. Data processing and analysis were performed with a combination of in-house developed codes and the PLS toolbox 8.8 (R8.8.1: Eigenvector Research, Inc., Manson, WA, USA). The images were arranged in a 3D array (cube) in which the x- and y-axes corresponded to the pixel coordinates while the *m*/*z* values registered in each pixel were reported along z-axes. The cube was transformed into a 2D matrix (D), where the x dimension is the number of pixels and y is the number of measured variables. All the pre-processing and algorithms were applied to the unfolded matrix D containing the TIC normalized spectra. Firstly, penalized asymmetric least squares (AsLS) smoothing was applied to correct the baseline and remove the interference using the following parameters: *p* = 0.005, λ = 108, with 20 iterations [52]. Then, the relevant peaks were discriminated from the noise using the median absolute deviation (MAD). A signal-to-noise threshold was set at 2.5MAD and only the intensities above the threshold were used for the analysis. Variables that presented signals of less than 35% pixels were substituted with the minimum values for those variables. This step reduced the positive ion mode variables to 23,694 and the negative ion mode variables to 30,327. Ultimately, the logarithm was calculated. The entire dataset was split into training and test sets. The images from patients 2, 3, 10, and 11 constitute the test set, the remaining data were used as a training set.

#### 3.11.2. Exploratory Analysis

The multivariate analysis was carried out considering objects identifiable within the image. In its simplest implementation, object classification relies on describing the tissue by the median spectrum of the pixels associated with it. Then the models were applied to the entire images for both training and test sets. Therefore, Principal component analysis (PCA) was carried out on the auto-scaled training set. Then, the obtained loadings were applied to the entire images of both training and test sets in order to build score maps.

#### 3.11.3. Partial Least Squares-Discriminant Analysis (PLS-DA)

Partial least squares-discriminant analysis (PLS-DA) is a supervised classification algorithm based on PLS regression [53]. Conversely to the regression approach, the Y variable is a binary-coded categorical vector for classification purposes. The PLS calculates a regression model relating the predictor matrix and Y. Then, classification is achieved by thresholding the values of the predicted response; several approaches are reported in the literature for such a purpose. In the present study, classification was achieved by applying linear discriminant analysis (LDA) to the predicted values of the response [54]. In order to select the optimal number of latent variables (nLVs) that maximized the accuracy and minimized the classification error, cross-validation (CV) was performed. Both images, pathological and healthy, collected from a specific patient were left out at each iteration. As support for further metabolic interpretation of the observed differences between healthy and pathological tissues, the values of the variable importance in projection (VIP) indices were calculated based on the model [55]. To evaluate the PLS-DA performances, the confusion matrix was calculated for each model in training, cross-validation, and external prediction (test), allowing the estimation of the main classification figures of merit:

○ Classification accuracy (or percent correct classification rate), which is defined as:

$$\mathrm{Accuracy}\;\left(\mathrm{CC}\%\right)=\frac{\mathrm{TP}+\mathrm{TN}}{\mathrm{TP}+\mathrm{FP}+\mathrm{TN}+\mathrm{FN}}$$

○ Sensitivity, i.e., the percent true positive rate:

$$\mathrm{Sensitivity}\;\left(\%\right)=\frac{\mathrm{TP}}{\mathrm{TP}+\mathrm{FN}}*100$$

○ Specificity, i.e., the true negative rate:

$$\mathrm{Specificity}\;\left(\%\right)=\frac{\mathrm{TN}}{\mathrm{TN}+\mathrm{FP}}*100$$

where TP, TN, FP, and FN are true positive, true negative, false positive, and false Negative values, respectively.

## 4. Conclusions

The present study allowed identifying the relevant metabolites of salivary gland tissues significantly associated with the presence of parotid neoplasms, taking advantage of MALDI-MSI technology combined with statistical approaches. The statistical model was able to correctly classify the Warthin tumor cases for both training and test sets, with sufficient accuracy (95%) considering the size of the pilot cohort (11 patients). The satisfying alignment between the multivariate analysis and the spatial segmentation highlighted the metabo-lipidomics signature of the tumor parotid tissues, which appears to be mainly correlated with the metabolism of membrane lipids as well as polar and non-polar metabolites involved in the signaling pathway and energy production of neoplastic cells. In particular, glycerophospholipids, glutamate metabolism, and nucleotides were sensibly increased in tumor regions, while the opposite was observed for sphingomyelins and triacylglycerols. These results need to be verified on a larger cohort of patients; however, they show the possibility of using MALDI-MSI-based metabolomics as a complementary diagnostic tool in routine fine-needle aspiration (FNA) and cytopathology in the diagnosis of salivary gland neoplasms [56].

## Figures and Tables

**Table 1:** Partial least squares-discriminant analysis (PLS-DA) of MALDI-positive and -negative TOF-MSI data. Classification results in training, leave-one-patient-out cross-validation, and test steps. The models were applied to the images. Sensitivity and specificity refer to the healthy classes. Abbreviations: CV, cross-validation; nVar, number of variables; nLVs, number of latent variables.

***MALDI-Positive TOF-MSI***								
**Range**	**nVar**	**nLVs**	**Accuracy (CC%)**		**Sensitivity (%)**		**Specificity (%)**	
***Training and cross-validation (CV)***								
			**Training**	**CV**	**Training**	**CV**	**Training**	**CV**
**Full**	23,694	3	100.00	100.00	100.00	100.00	100.00	100.00
**VIP**	441	1	100.00	100.00	100.00	100.00	100.00	100.00
***Test set***								
**Full**			69.00		76.31		64.44	
**VIP**			69.00		76.31		64.44	
***MALDI-Negative TOF-MSI***								
**nLVs**	**nLVs**	**nLVs**	**Accuracy (CC%)**		**Sensitivity (%)**		**Specificity (%)**	
***Training and cross-validation (CV)***								
			**Training**	**CV**	**Training**	**CV**	**Training**	**CV**
**Full**	30,327	3	100.00	92.68	100.00	100.00	100.00	85.71
**VIP**	245	3	100.00	100.00	100.00	100.00	100.00	100.00
***Test set***								
**Full**			68.04		90.68		53.72	
**VIP**			68.04		90.68		53.72	

**Table 2:** List of statistically relevant annotated metabolites derived from PLS-DA and/or a spatial segmentation analysis. Detailed parameters for the tentative annotation are reported in Tables S2 and S3. (↑, Up-regulation in Tumor; ↓, Down-regulation in Tumor).

Compound	*m/z*	Adduct	Molecular Formula	VIP Score	Pearson Correl. Coeff. (*r*)	Modulation in Tumor
CAR 16:0	400.3	[M+H]^+^	C_23_H_45_NO_4_	2.580		↓
Cer 42:2;O	632.6	[M+H]^+^	C_42_H_81_NO_2_	2.188		↑
DG O-36:4	603.5	[M+H]^+^	C_39_H_70_O_4_	2.001		↓
LPC 15:0	480.3	[M-H]^−^	C_23_H_48_NO_7_P	2.172		↓
LPC 16:0	496.3	[M+H]^+^	C_24_H_50_NO_7_P	2.078		↓
PC 30:0	744.4	[M+K]^+^	C_38_H_76_NO_8_P		0.563	↑
PC 32:0	772.5	[M+K]^+^	C_43_H_82_NO_8_P	2.032	0.702	↑
PC 32:1	770.5	[M+K]^+^	C_40_H_78_NO_8_P	2.007		↑
PC 34:1	798.5	[M+K]^+^	C_42_H_82_NO_8_P		0.687	↑
PC 34:3	756.5	[M+H]^+^	C_42_H_78_NO_8_P		0.622	↑
PC 32:2 or PE 35:2	730.5	[M+H]^+^	C_40_H_76_NO_8_P	2.036		↑
PC 33:2 or PE 36:2	744.5	[M+H]^+^	C_41_H_78_NO_8_P		0.563	↑
PC 34:2	758.5	[M+K]^+^	C_42_H_80_NO_8_P	2.282	0.679	↓
PC 38:3	812.6	[M+H]^+^	C_46_H_86_NO_8_P		0.563	↑
PC 21:1	578.4	[M+H]^+^	C_29_H_56_NO_8_P	2.169		↑
PC 25:1;O	650.4	[M+H]^+^	C_33_H_64_NO_9_P		0.503	↓
PC O-32:2	551.5	[M+H]^+^	C_35_H_66_O_4_	3.525		↓
PC O-42:6	870.6	[M+Na]^+^	C_50_H_90_NO_7_P		0.519	↓
PC O-44:5	900.7	[M+Na]^+^	C_52_H_96_NO_7_P	2.086		↓
PE 34:0	718.5	[M-H]^−^	C_39_H_78_NO_8_P		0.592	↑
PE 36:3	742.5	[M+H]^+^	C_41_H_76_NO_8_P		0.610	↑
PE 40:2	800.5	[M+H]^+^	C_45_H_86_NO_8_P		0.742	↑
PE 42:7	818.4	[M+H]^+^	C_47_H_80_NO_8_P	2.198		↑
PE O-38:6	750.5	[M+H]^+^	C_43_H_76_NO_7_P	2.045	0.681	↓
PS O-30:2	712.4	[M+Na]^+^	C_36_H_68_NO_9_P		0.633	↓
PS 36:1	524.3	[M-H]^−^	C_24_H_48_NO_9_P		0.552	↑
SM 44:1;O2	881.6	[M+K]^+^	C_49_H_99_N_2_O_6_P	4.523		↓
SM 41:2;O2	821.6	[M+Na]^+^	C_46_H_91_N_2_O_6_P	2.425		↑
SM 43:2;O2	827.6	[M+H]^+^	C_48_H_95_N_2_O_6_P	2.079		↓
SM 36:1;O2	731.6	[M+H]^+^	C_41_H_83_N_2_O_6_P	2.011	0.667	↓
SM 38:1;O2	781.5	[M+Na]^+^	C_43_H_87_N_2_O_6_P	2.617	0.681	↓
SM 34:2;O2	723.5	[M+Na]^+^	C_39_H_77_N_2_O_6_P	2.055	0.680	↓
SM 38:0;O2	761.5	[M+H]^+^	C_43_H_89_N_2_O_6_P	2.001		↓
TG 54:4	883.7	[M+H]^+^	C_57_H_102_O_6_	2.092		↓
TG 53:2	895.7	[M+Na]^+^	C_56_H_104_O_6_	2.149		↓
TG 52:2	881.7	[M+Na]^+^	C_55_H_102_O_6_	2.551		↓
TG 54:3	907.7	[M+Na]^+^	C_57_H_104_O_6_	2.198		↓
TG 56:5	909.7	[M+H]^+^	C_59_H_104_O_6_	2.001		↓
TG 58:9	929.7	[M+H]^+^	C_61_H_100_O_6_	2.265		↓
FA 18:2	279.2	[M-H]^−^	C_18_H_32_O_2_		0.529	↓
FA 20:4	303.2	[M-H]^−^	C_20_H_32_O_2_		0.544	↓
FA 22:6	327.2	[M-H]^−^	C_22_H_32_O_2_		0.519	↓
FA 18:0	283.2	[M-H]^−^	C_18_H_36_O_2_	2.023		↓
Glutamine	145.0	[M-H]^−^	C_5_H_10_N_2_O_3_		0.662	↑
Glutamate	146.0	[M-H]^−^	C_5_H_9_NO_4_		0.521	↑
ADP	426.0	[M-H]^−^	C_10_H_15_N_5_O_10_P_2_	2.034		↑
AMP	346.0	[M-H]^−^	C_10_H_14_N_5_O_7_P	2.049	0.623	↑
UMP	323.0	[M-H]^−^	C_9_H1_3_N_2_O_9_P	2.089		↑
GMP	362.0	[M-H]^−^	C_10_H_14_N_5_O_8_P		0.521	↑
Xanthine	151.0	[M-H]^−^	C_5_H_4_N_4_O_2_	2.013		↑
Hypoxanthine	135.0	[M-H]^−^	C_5_H_4_N_4_O	2.038	0.628	↑
Cytidine	242.0	[M-H]^−^	C_9_H_13_N_3_O_5_		0.553	↑
Aspartate	132.0	[M-H]^−^	C_4_H_7_NO_4_	2.002	0.563	↑

**Table 3:** Demographic and clinical data of the patients.

Parotid Tumor Subjects	
Age, median (range)	64 (43–78)
Sex (M/F)	3/8
Tumor histological type (patient classification number):	Warthin Tumor (1, 3, 4, 7, 8, 9, 10)
	Pleomorphic adenoma (5)
	Chronic sialoadenitis (2, 6, 11)

## Data Availability

The data that support the findings of this study are available from the corresponding author, P.C., upon reasonable request due to restrictions on privacy or ethical.

## Supplementary Materials

The following supporting information can be downloaded at: https://www.mdpi.com/article/10.3390/metabo12060530/s1, Section S1: Polar and non-polar metabolites extraction, UHPLC–HRMS/MS conditions, and data processing; Figure S1: Histopathology of parotid gland tumor tissues at low and high magnification; Figure S2: Score images for the MALDI-positive TOF-MSI dataset. Loadings obtained from PCA were applied to the images of the training set. PC2 and PC4 score images have been reported and compared with the histological images; Figure S3: Score images for the MALDI-positive TOF-MSI dataset. Loadings obtained from PCA were applied to the images of the test set. PC2 and PC4 score images have been reported and compared with the histological images; Figure S4: Score images for the MALDI-negative TOF-MSI dataset. Loadings obtained from object-level PCA were applied to the images of the training set. PC3 and PC4 score images have been reported and compared with the histological images; Figure S5: Score images for the MALDI-negative TOF-MSI dataset. Loadings obtained from object-level PCA were applied to the images of the test set. PC3 and PC4 score images have been reported and compared with the histological images; Figure S6: Prediction map of the training set of PLS-DA analysis for the MALDI-positive TOF-MSI analysis using the entire variable range. The percentage of correctly classified pixels is reported for each image; Figure S7: Prediction map of test set of PLS-DA analysis for the MALDI-positive TOF-MSI analysis using the entire variable range. The percentage of correctly classified pixels is reported for each image; Figure S8: Prediction map of training set of PLS-DA analysis for the MALDI-positive TOF-MSI analysis using the variable with VIP scores greater than 2. The percentage of correctly classified pixels is reported for each image; Figure S9: Prediction map of test set of PLS-DA analysis for the MALDI-positive TOF-MSI analysis using the variable with VIP scores greater than 2. The percentage of correctly classified pixels is reported for each image; Figure S10: Prediction map of training set of PLS-DA analysis for the MALDI-negative TOF-MSI analysis using the entire variable range. The percentage of correctly classified pixels is reported for each image; Figure S11: Prediction map of test set of PLS-DA analysis for the MALDI-negative TOF-MSI analysis using the entire variable range. The percentage of correctly classified pixels is reported for each image; Figure S12: Prediction map of training set of PLS-DA analysis for the MALDI-negative TOF-MSI analysis using the variable with VIP scores greater than 2. The percentage of correctly classified pixels is reported for each image; Figure S13: Prediction map of test set of PLS-DA analysis for the MALDI-negative TOF-MSI analysis using the variable with VIP scores greater than 2. The percentage of correctly classified pixels is reported for each image; Table S1: List of lipids and polar metabolites detected in MALDI-MSI experiments and tentatively identified by MALDI-FTICR and/or LC-HRMS/MS; Table S2: Additional RP-UHPLC-TIMS-MS to enforce the annotation of the statistical relevant lipids derived from PLS-DA and/or spatial segmentation analysis. Lipids were reported with long name nomenclature if MS/MS spectra contained information about fatty acyl composition, otherwise a short name nomenclature was adopted; Table S3: Additional UHPLC–HRMS/MS to enforce the annotation of the statistical relevant non-polar and polar metabolites derived from PLS-DA and/or spatial segmentation analysis.
