# Supplementary material for: MALDI Mass Spectrometry Imaging Highlights Specific Metabolome and Lipidome Profiles in Salivary Gland Tumor Tissues
Source: Metabolites. 2022 Jun 8;12(6):530. doi: 10.3390/metabo12060530 (PMC9228942; doi:10.3390/metabo12060530)
Supplement: Supplementary file 1 [file metabolites-12-00530-s001.zip › metabolites-1739705-supplementary.pdf]

Supplementary materials for the manuscript

MALDI Mass Spectrometry Imaging highlights specific metabolome and lipidome profiles in salivary gland tumor tissues

Eduardo Sommella [1,†], Emanuela Salviati [1,†], Vicky Caponigro [1], Manuela Grimaldi [1], Simona Musella [1], Alessia Bertamino [1], Luigi Cacace [2], Remo Palladino [2], Giuseppe Di Mauro [2], Federico Marini [3], Anna Maria D'Ursi [1] and Pietro Campiglia [1,*]

1 Department of Pharmacy, University of Salerno, Via Giovanni Paolo II, 132, 84084 Fisciano, Italy; esommella@unisa.it (E.S.); esalviati@unisa.it (E.S.); vcaponigro@unisa.it (V.C.); magrimaldi@unisa.it (M.G.); smusella@unisa.it (S.M.); abertamino@unisa.it (A.B.); dursi@unisa.it (A.M.D.)

2 U.O.C Clinical Pathology D.E.A. III Umberto I, Viale S. Francesco D'Assisi, 84014 Nocera Inferiore, Italy; cacace10luigi@gmail.com (L.C.); remo.palladino@tiscali.it (R.P.); dr.giuseppedimauro@gmail.com (G.D.M.)

3 Department of Chemistry, University of Rome "La Sapienza", Piazzale Aldo Moro 5, 00185 Rome, Italy; federico.marini@uniroma1.it

* Correspondence: pcampiglia@unisa.it; Tel.: +39-089-969242

† These authors contributed equally to this work.

Section S1. Polar and non-polar metabolites extraction, UHPLC-HRMS/MS conditions and data processing.

Despite FT-ICR provided ultra-high mass accuracy (average −0.14 ± 1.32 ppm), this data alone can not differentiate the large amount of isomers occurring in biological samples, and thus additional information, such as MS/MS fragmentation pattern comparison against a reference spectra, is mandatory for reliable metabolite annotations [24]. For this reason, the structural confirmation of many of the mass-matched compounds detected was performed "off tissue" by two UHPLC-HRMS/MS distinct methods for lipids and polar metabolite extracts, RP-UHPLC-TIMS- Q-TOF and HILIC-UHPLC-QExactive-Orbitrap-MS, respectively. The largest part of metabolites was annotated with MSI level 2 with accurate mass/spectral similarity/isotopic fine structure. Where MS/MS data were not available, they were tentatively assigned to chemical classes and reported with MSI level 3 [25].

Polar metabolites and lipids were extracted following the Matyash protocol [48] with slight modifications. Briefly, 5 mg of lyophilized tissues were treated with 225 µL of ice-cold MeOH and were vortexed for 30 s. Subsequently 750 µL of ice-cold MTBE were added and the solution was mixed in a thermomixer® (Eppendorf) for 6 min at 4°C. Subsequently 188 µL of H_2O were added and samples were vortexed for 20 s and finally centrifuged at 14 680 rpm, for 5 min at 4°C for phase separation. The two phases were recovered separately and dried under nitrogen. The non-polar extract was solubilized in 400 µL of 5 mM CH_3COONH_4 in MeOH/DCM (90/10 v/v), while the polar extract was solubilized in 200 µL of 1:1 ACN/H_2O + 0.1% HCOOH. A pooled Quality Control (QC) sample was prepared by pooling an aliquot of tissue extract from each sample.

The analysis of polar sample analyses was performed on a Thermo Ultimate RS 3000 coupled online to a Q-Exactive hybrid quadrupole Orbitrap mass spectrometer (Thermo Fisher Scientific, Bremen, Germany) equipped with a heated electrospray ionization probe (HESI II). The MS was calibrated by Thermo calmix Pierce™ calibration solutions in both polarities. Separation was performed in HILIC mode, with an Acquity BEH Amide (150 × 2.1 mm; 1.7 µm) protected with a VanGuard amide precolumn (5 × 2.1 mm; 1.7 µm) (Waters, Milford, MA, U.S.A). The column temperature was set at 45°C, and the flow rate was 0.350 mL/min. The mobile phase was (A): 5 mM CH_3COONH_4 in H_2O/ACN (95:5 v/v) + 0.1% CH_3COOH (v/v) and (B): 5 mM CH_3COONH_4 in H_2O/ACN (5:95 v/v) + 0.1% CH_3COOH (v/v). The following gradient was employed: 0 min, 99% B, 0.01-1 min, 99% B, 1.01-12 min, 99.01-20% B, 12.01-13 min, 20% B, returning to 99% in 0.1 min, 3 µL and 5 µL were injected for ESI^+ and ESI^- analysis, respectively. Full MS (80-800 m/z) and data-dependent MS/MS were performed at a resolution of 35,000 and 15,000 FWHM respectively, normalized collision energy (NCE) values of 10, 20, and 30 were used. Source parameters: Sheath gas pressure, 50 arbitrary units; auxiliary gas flow, 13 arbitrary units; spray voltage, +3.5 kV, -2.8 kV; capillary temperature, 310°C; auxiliary gas heater temperature, 300°C. Three replicate of each sample were performed in each polarity, QC was randomly inserted in the batch to monitor system stability over time.

Lipid analysis was performed on Thermo Ultimate RS 3000 coupled online to a Trapped Ion-Mobility Time-of-Flight (timsTOF) Pro mass spectrometer ((Bruker Daltonik, Bremen, Germany) equipped with an electrospray ionization source (ESI). For the chromatographic separation, an Acquity UPLC CSH C18 column (100 × 2.1 mm; 1.7 µm) protected with a VanGuard CSH precolumn (5 × 2.1 mm; 1.7 µm) (Waters, Milford, MA, U.S.A) was employed. The column temperature was set at 55°C, a flow rate of 0.4 mL/min was used, mobile phase consisted of (A): ACN/H_2O with 10 mM CH_3COONH_4 and 0.1 % HCOOH 60:40 (v/v) and (B): IPA/ACN with 0.1 % HCOOH 90:10 (v/v). The following gradient has been used: 0 min, 40% B, 2 min, 43% B, 2.10 min, 50% B, 10 min, 60% B, 12 min,75% B, 13 min, 99% B hold for 2 min, returning to 40% in 0.1 min.

Q-Exactive MS/MS data analysis was performed with MS-DIAL v4.48 (http://prime.psc.riken.jp/compms/msdial/main.html). Thermo RAW. data files were converted to ABF format using Reifycs Abf (Analysis Base File) converter, subsequently alignment of Profile Q-Exactive files was performed with MS tolerance set at 0.01 for MS and 0.025 for MS/MS with retention time tolerance of 0.1 min. Minimum peak height for detection was set to 10000 amplitude value. Lipid identification was performed with internal Lipidblast library while metabolites with internal libraries ESI POS and ESI NEG All_Public_MS/MS. All metabolites annotations were based on mass accuracy, isotopic pattern and spectral matching, rev.dot product, score cut off was 70%. All

reported spectral matches were manually revised for correct assignment, adducts with sodium, formate and acetate were allowed for adduct correction. The alignment was performed only on matched MS/MS metabolites blank corrected.

TimsTOF-MS data analysis was performed with MetaboScape 2021 (Bruker) employing a feature finding algorithm (T-Rex 4D) that automatically extracts buckets from raw files. At the beginning of each LC-MS run, a mixture (1:1 v/v) of 10mM sodium formate calibrant solution and ESI-L Low Concentration Tuning Mix was injected to recalibrate, respectively, the mass and mobility data. Feature detection was set to 500 and 250 counts for positive and negative modes. The minimum number of data points in the 4D-TIMS space was set to 100, and recursive feature extraction was used (75 points). Lipid annotation was performed first with a rule-based annotation, based on characteristic fragments and their intensity in acquired MS/MS spectra, and, subsequently, using the LipidBlast spectral library of MS DIAL (http://prime.psc.riken.jp/compms/msdial/main.html) with the following parameters: Mass accuracy: narrow 2 ppm, wide 10 ppm; mSigma: narrow 30, wide 250, MS/MS score: narrow 800, wide 150. Collision Cross-Section (CCS)%: narrow 2, wide 3.5. The spectra were processed in positive mode using $[M+H]^+$, $[M+Na]^+$, $[M+K]^+$, $[M+H–H_2O]^+$ and $[M+NH_4]^+$ ions, while $[M–H]^-$, $[M+Cl]^-$, $[M+CH_3COO]^-$ and $[M–H_2O]^-$ in negative mode. CCS values were compared with those predicted by CCSbase platform (https://ccsbase.net/predictions), the assignment of the molecular formula was performed for the detected features using Smart Formula™ (SF). Each lipid feature was manually curated following Lipidomics Standard Initiative (LSI) guidelines (https://lipidomics-standards-initiative.org/guidelines/lipid-species-identification/general-rules). LipidCreator tool (https://lifs-tools.org/lipidcreator.html) extension in the Skyline (https://skyline.ms/project/home/begin.view) as used for in silico comparison of specific product ions for manual MS/MS curation.

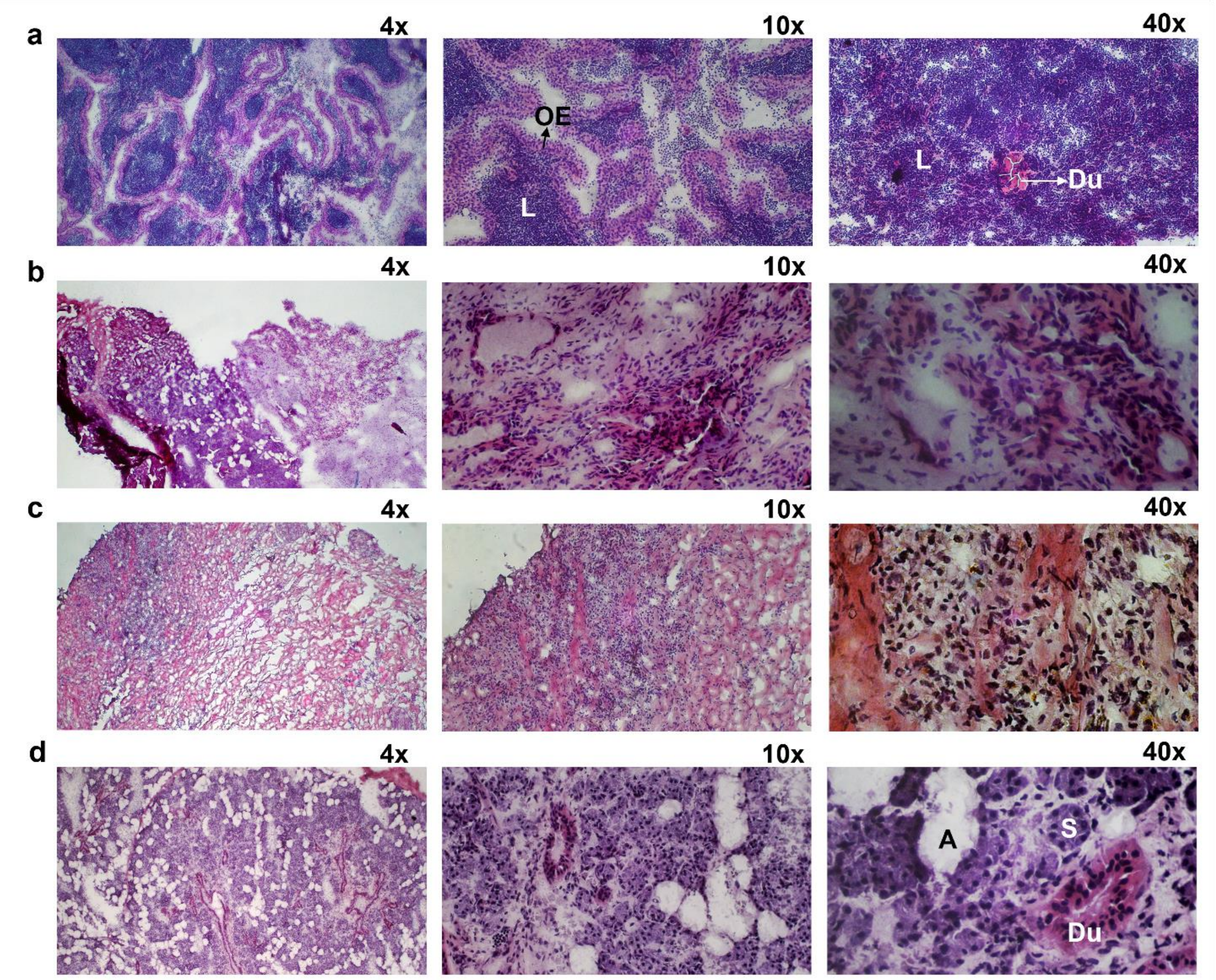

Figure S1. Histopathology of parotid gland tumor tissues at low and high magnification. (**a**) The Warthin tumor tissue shows variable proportions of papillary-cystic structures lined with a bilayer of epithelial cells consisting of oncocytic columnar cells (oncocytic epithelial cells, **OE**) with underlying discontinuous basal cells and resting on dense lymphoid stroma (**L**) with variable germinal centers.

Lymphocytes are typically small mature and have high nucleus-cytoplasm ratio with deeply stained nucleus and a relatively small amount of cytoplasm. Oncocytes show densely granular cytoplasm (stuffed with mitochondria on electron microscopy), centrally located nuclei and small nucleoli. (**b**) Pleomorphic adenoma consists of epithelial, myoepithelial and stromal components. The histopathologic appearance shows a tumor with a lobulated growth pattern with tubular and acinar structures formed by the epithelial component mixed with myoepithelial cells in a background of myxoid stroma. (**c**) The histopathological area of the chronic sialoadenitis shows various degrees of acinar destruction, fibrosis and chronic and active periductal inflammation with lymphoid aggregates and focal granulomatous reaction. (**d**) The normal parotid gland tissue is mainly characterized by secretory acini, serous and mucous (**S**) and intercalated ducts (**D**), which are lined by simple low cuboidal epithelium and surrounded by myoepithelial cells. The glands are divided into lobules by connective tissue sept and there are adipocytes (**A**) between acini.

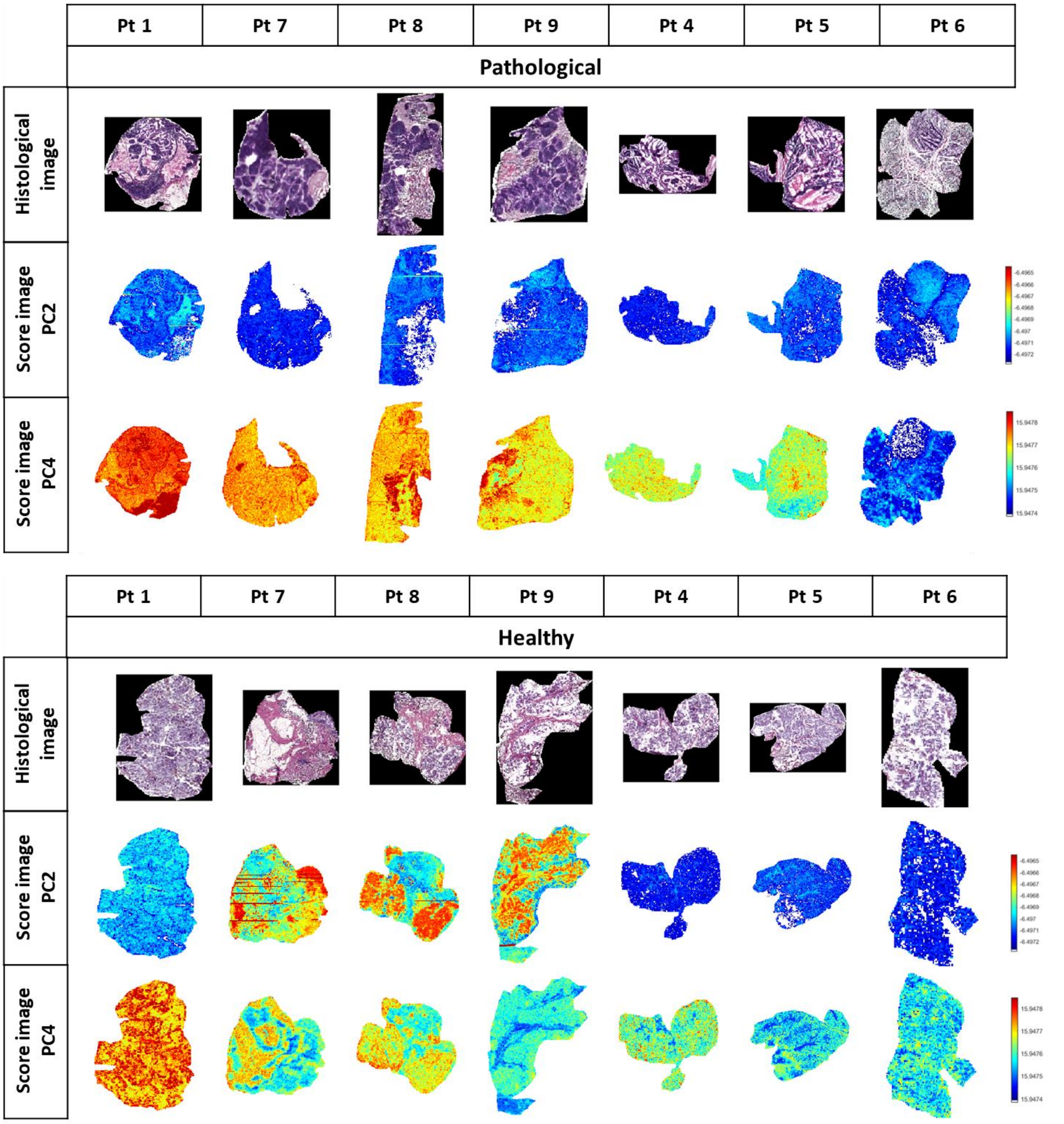

Figure S2. Score images for the MALDI positive TOF-MSI dataset. Loadings obtained from PCA have been applied to the entire images of the training set. PC2 and PC4 score images have been reported and compared with the histological images. Abbreviation: Pt, Patient.

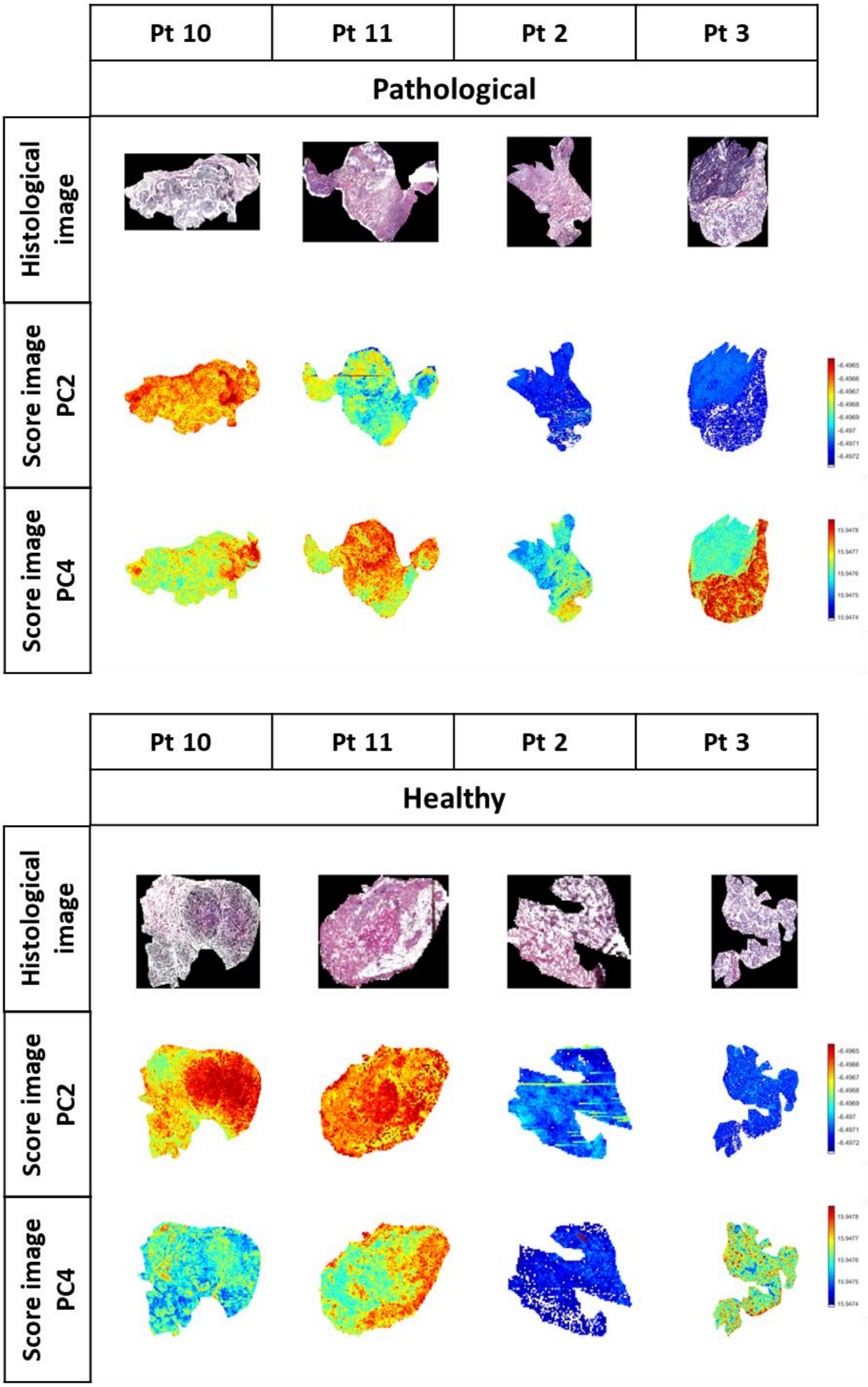

Figure S3. Score images for the MALDI positive TOF-MSI dataset. Loadings obtained from PCA have been applied to the entire images of the test set. PC2 and PC4 score images have been reported and compared with the histological images. Abbreviation: Pt, Patient.

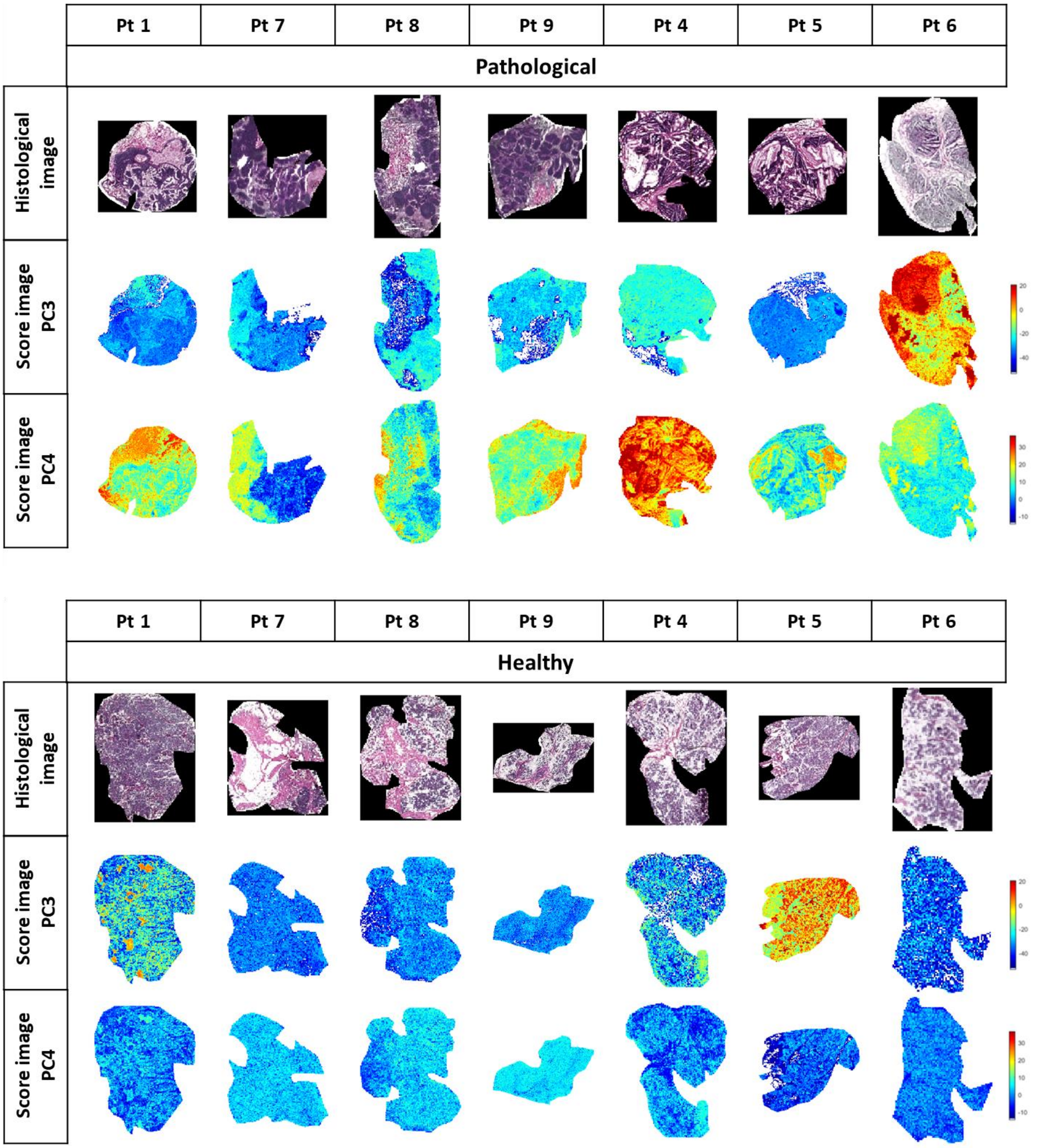

Figure S4. Score images for the MALDI negative TOF-MSI dataset. Loadings obtained from object level PCA have been applied to the entire images of the training set. PC3 and PC4 score images have been reported and compared with the histological images. Abbreviation: Pt, Patient.

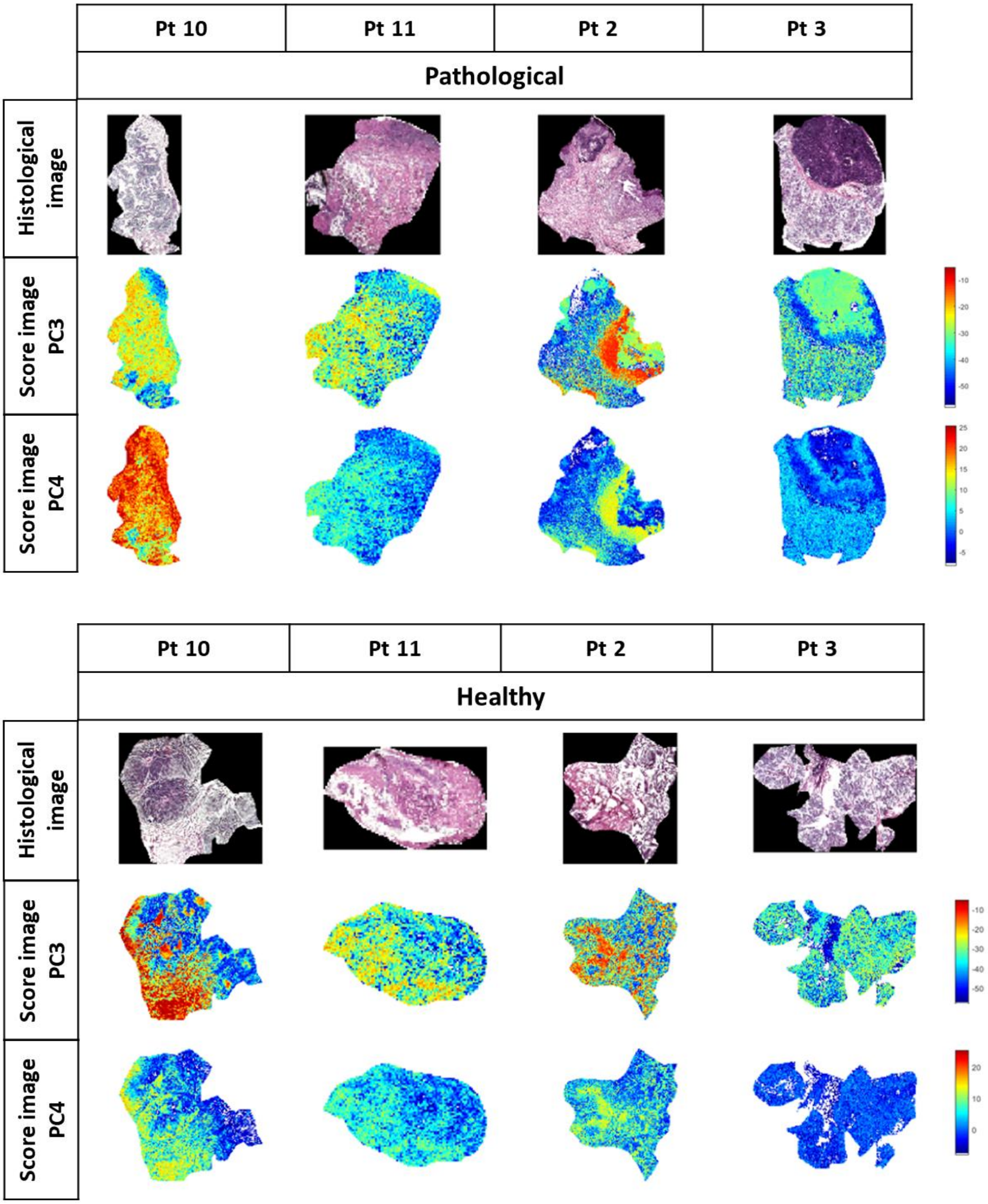

Figure S5. Score images for the MALDI negative TOF-MSI dataset. Loadings obtained from object level PCA have been applied to the entire images of the test set. PC3 and PC4 score images have been reported and compared with the histological images. Abbreviation: Pt, Patient.

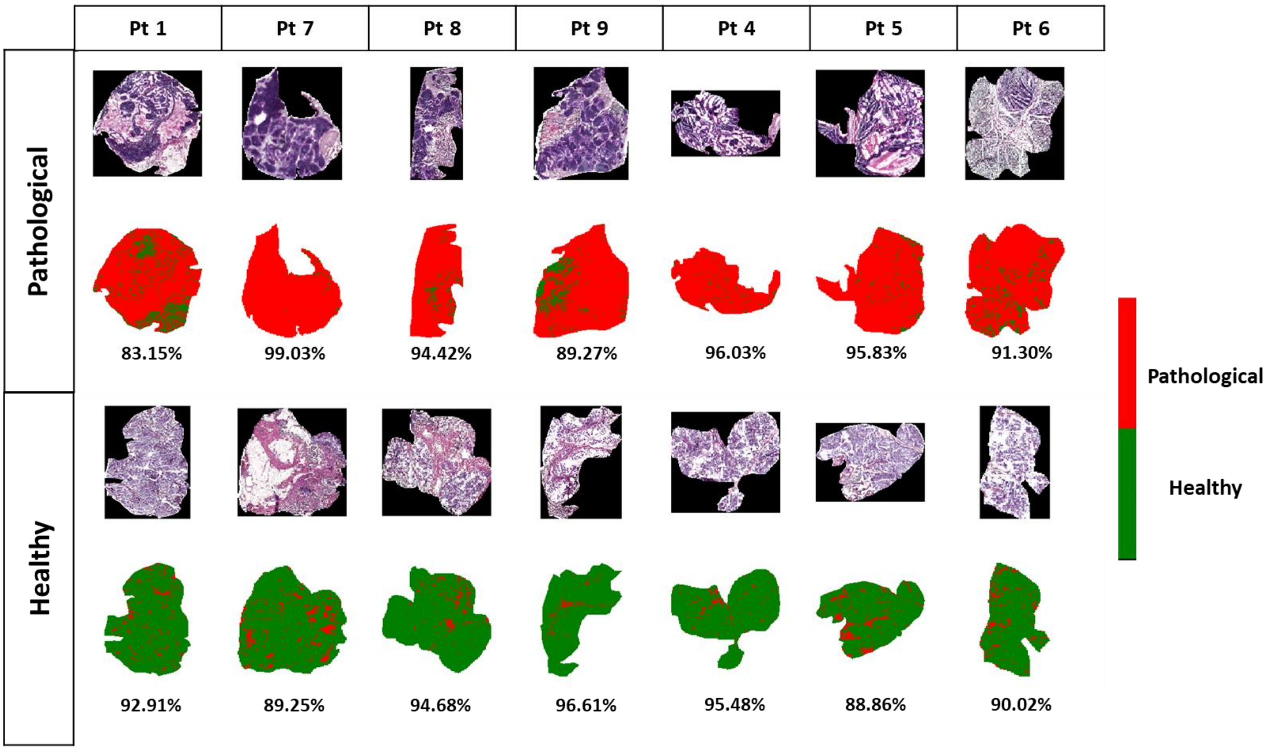

Figure S6. Prediction map of training set of PLS-DA analysis for the MALDI positive TOF-MSI analysis using the entire variable range. The percentage of correct classified pixels is reported for each image. Abbreviation: Pt, Patient.

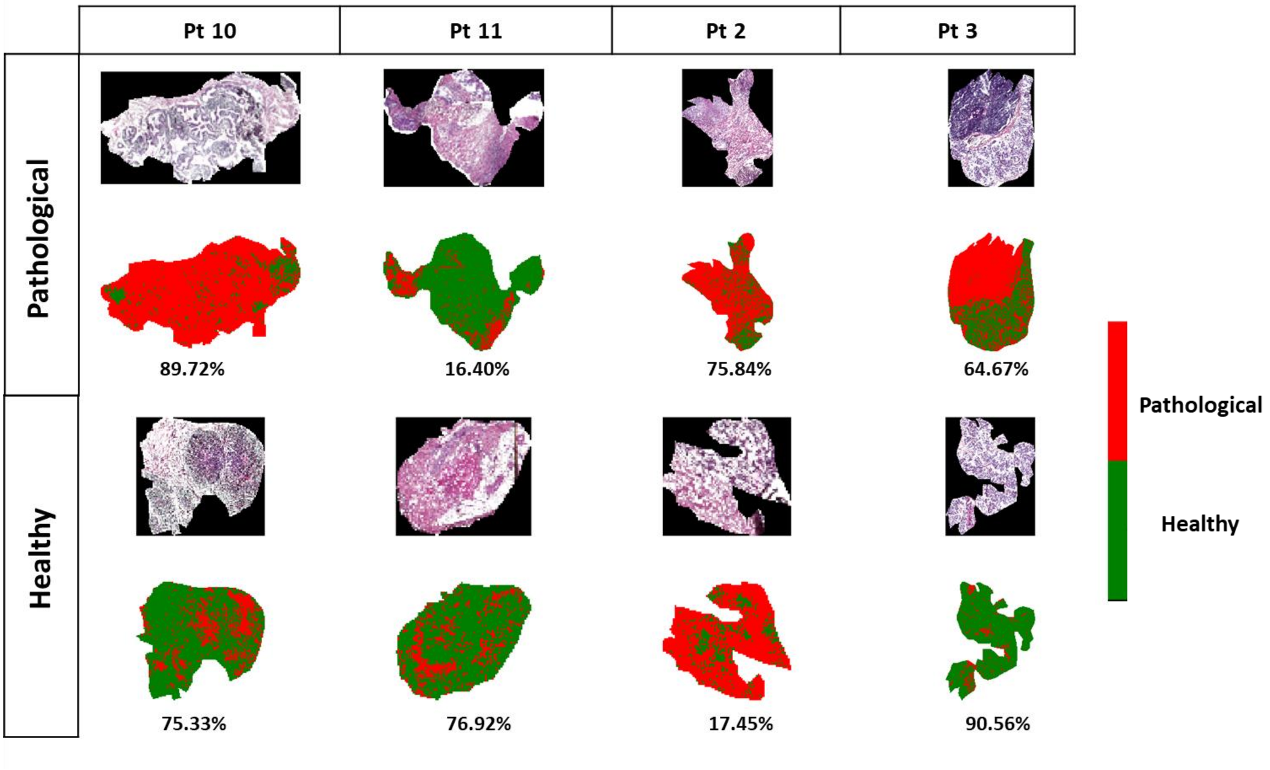

Figure S7. Prediction map of test set of PLS-DA analysis for the MALDI positive TOF-MSI analysis using the entire variable range. The percentage of correct classified pixels is reported for each image. Abbreviation: Pt, Patient.

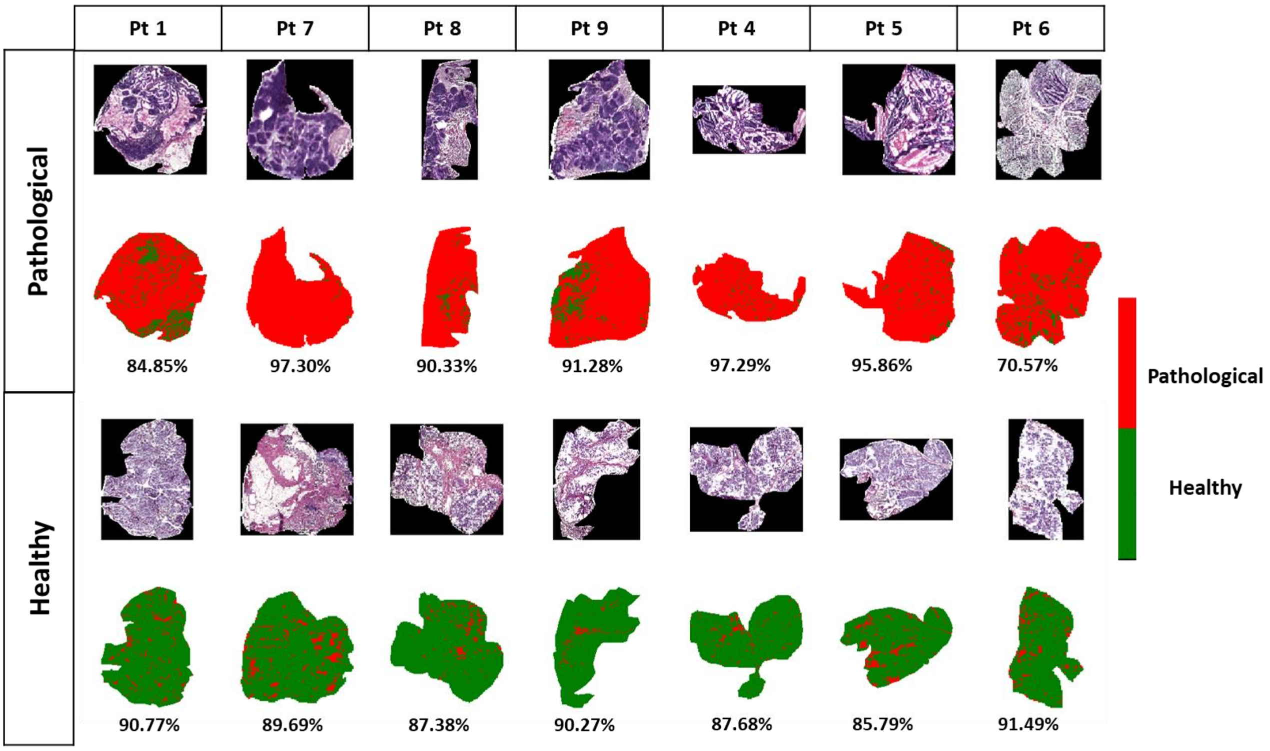

Figure S8. Prediction map of training set of PLS-DA analysis for the MALDI positive TOF-MSI analysis using the variable with VIP scores greater than 2. The percentage of correct classified pixels is reported for each image. Abbreviation: Pt, Patient.

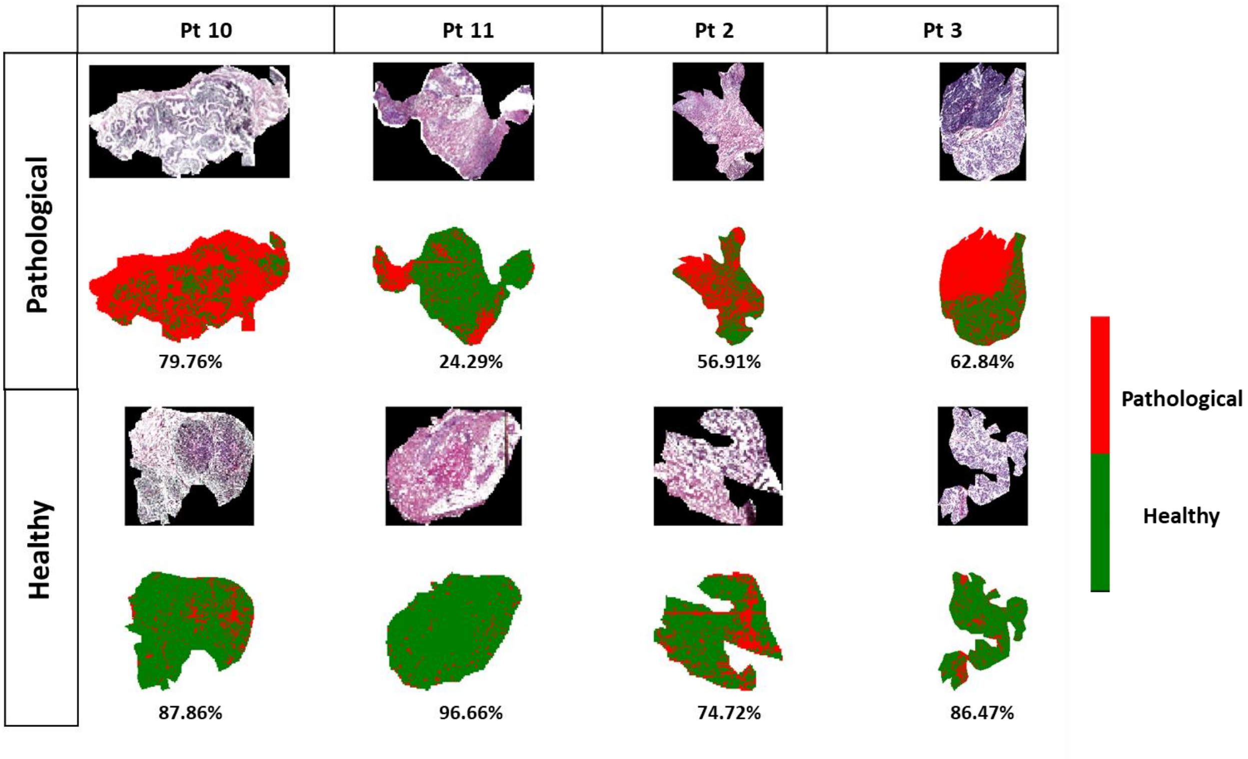

Figure S9. Prediction map of test set of PLS-DA analysis for the MALDI positive TOF-MSI analysis using the variable with VIP scores greater than 2. The percentage of correct classified pixels is reported for each image. Abbreviation: Pt, Patient.

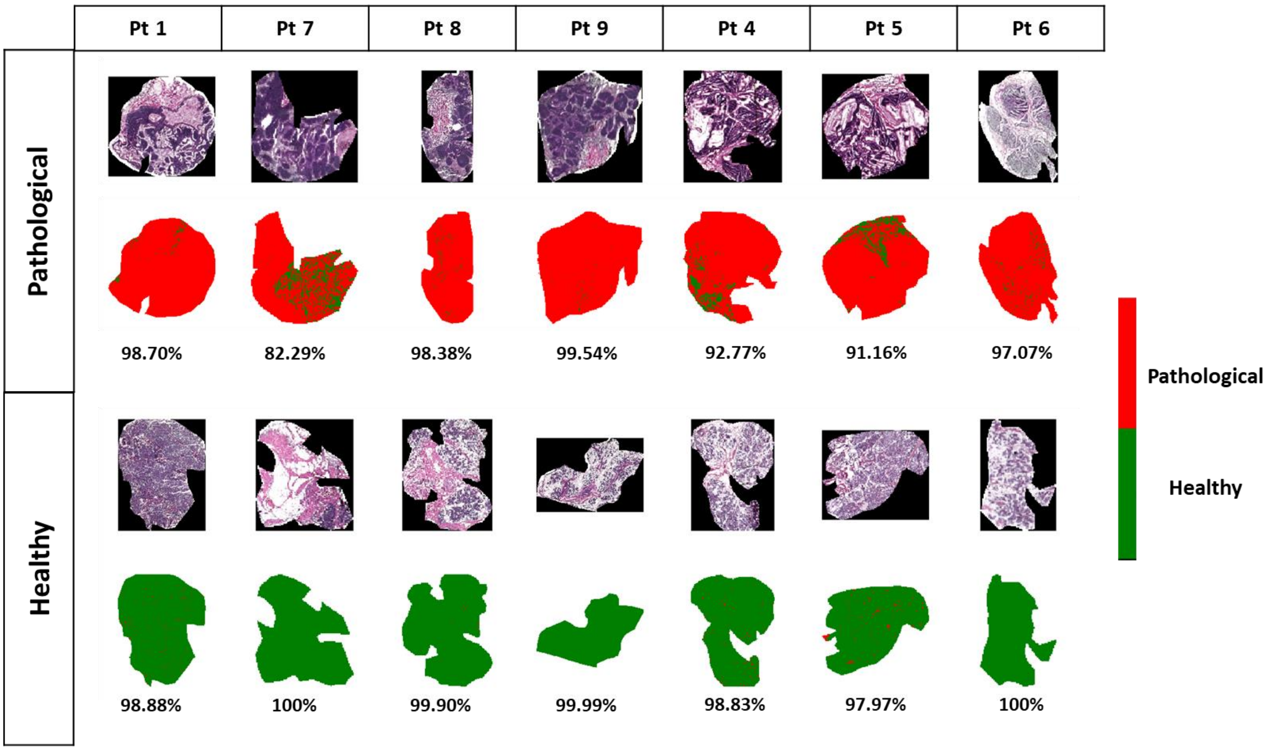

Figure S10. Prediction map of training set of PLS-DA analysis for the MALDI negative TOF-MSI analysis using the entire variable range. The percentage of correct classified pixels is reported for each image. Abbreviation: Pt, Patient.

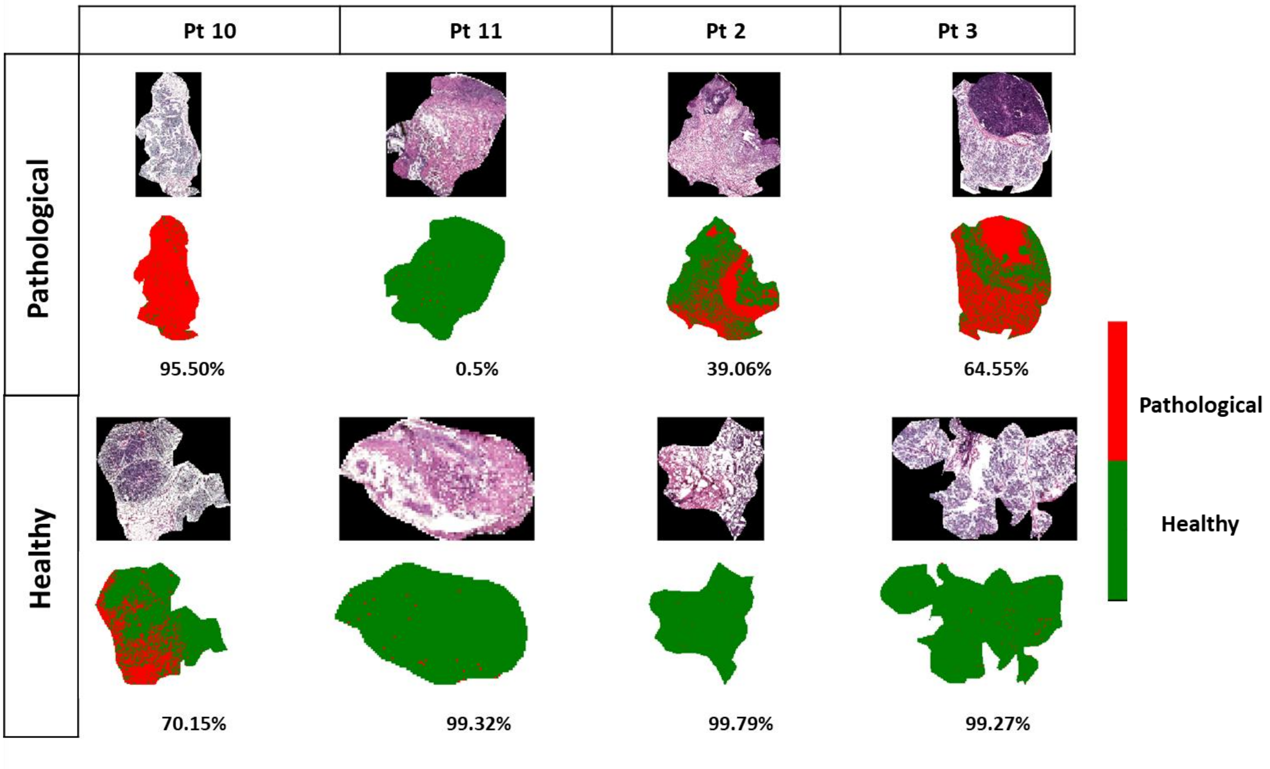

Figure S11. Prediction map of test set of PLS-DA analysis for the MALDI negative TOF-MSI analysis using the entire variable range. The percentage of correct classified pixels is reported for each image. Abbreviation: Pt, Patient.

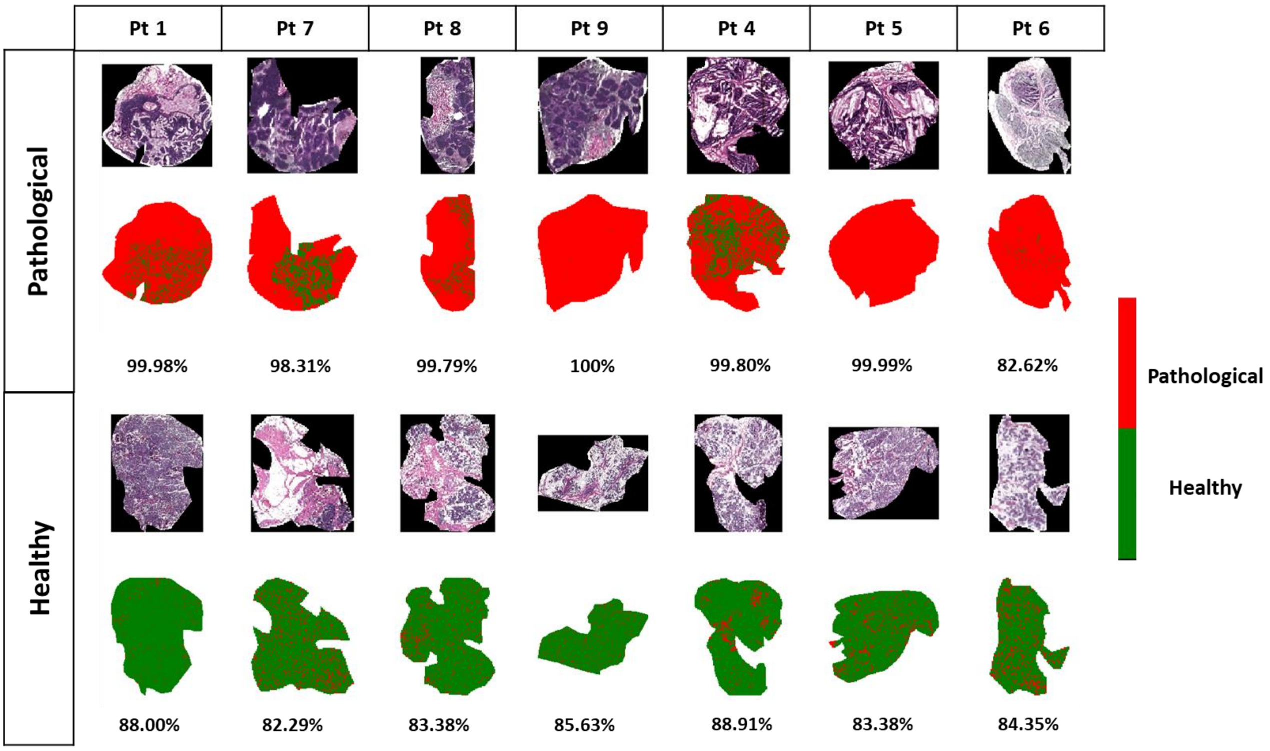

Figure S12. Prediction map of training set of PLS-DA analysis for the MALDI negative TOF-MSI analysis using the variable with VIP scores greater than 2. The percentage of correct classified pixels is reported for each image. Abbreviation: Pt, Patient.

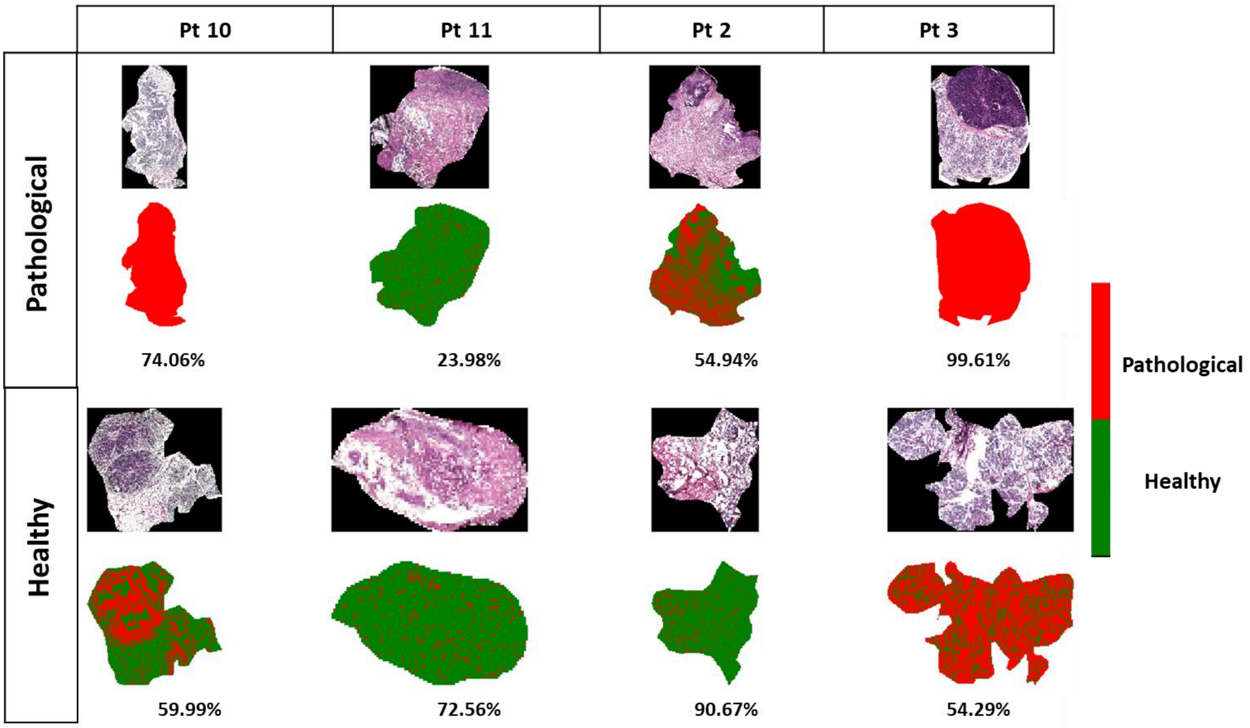

Figure S13. Prediction map of test set of PLS-DA analysis for the MALDI negative TOF-MSI analysis using the variable with VIP scores greater than 2. The percentage of correct classified pixels is reported for each image. Abbreviation: Pt, Patient.

Table S1. List of lipids and polar metabolites detected in MALDI-MSI experiments and tentatively identified by MALDI-FTICR and/or LC-HRMS/MS.

Lipids							
**Compound**	**Biochemical class/Subclass**	**RapifleX® *m/z***	**SolariX® *m/z***	**Error (ppm)**	**Adduct (*)**	**Molecular Formula**	**MS Identification Level (**)**
FAHFA 37:4;O	Fatty Acyls [FA]	575.501	575.50118	-3.832	**[M+H]⁺**, [M+Na] ⁺	$C_{37}H_{66}O_4$	3
FOH 37:4;O3	Fatty Acyls [FA]	577.542	577.51697	-3.562	**[M+H]⁺**, [M+Na]⁺	$C_{37}H_{68}O_4$	3
FA 20:1	Fatty Acyls [FA]	311.314	311.29443	-0.203	**[M+H]⁺**	$C_{20}H_{38}O_2$	2
FA 20:4	Fatty Acyls [FA]	389.319	389.30501	-0.124	**[M+H]⁺**	$C_{25}H_{40}O_3$	2
CAR 16:1	Fatty Acyls [FA]	398.341	398.32654	-0.045	**[M+H]⁺**	$C_{23}H_{43}NO_4$	3
CAR 18:1	Fatty Acyls [FA]	426.354	426.35782	-0.022	**[M+H]⁺**	$C_{25}H_{47}NO_4$	2
CAR 14:0	Fatty Acyls [FA]	372.310	372.31087	0.005	**[M+H]⁺**	$C_{21}H_{41}NO_4$	3
FA 19:1;O	Fatty Acyls [FA]	313.207	313.27372	0.006	**[M+H]⁺**	$C_{19}H_{36}O_3$	3
CAR 16:0;O	Fatty Acyls [FA]	416.356	416.33711	0.023	**[M+H]⁺**	$C_{23}H_{45}NO_5$	3
FA 21:1:O	Fatty Acyls [FA]	341.331	341.30506	0.073	**[M+H]⁺**	$C_{21}H_{40}O_3$	3
CAR 14:0;O	Fatty Acyls [FA]	388.313	388.30578	0.074	**[M+H]⁺**	$C_{21}H_{41}NO_5$	3
CAR 20:1	Fatty Acyls [FA]	454.397	454.38910	0.129	**[M+H]⁺**	$C_{27}H_{51}NO_4$	3
CAR 18:0	Fatty Acyls [FA]	428.395	428.37347	0.135	**[M+H]⁺**	$C_{25}H_{49}NO_4$	3
CAR 16:0	Fatty Acyls [FA]	400.352	400.34217	0.179	**[M+H]⁺**	$C_{23}H_{45}NO_4$	2
CAR 18:1;O	Fatty Acyls [FA]	442.357	442.35275	0.187	**[M+H]⁺**	$C_{25}H_{47}NO_5$	3
FA 20:4;O	Fatty Acyls [FA]	321.253	321.24251	0.266	**[M+H]⁺**	$C_{20}H_{32}O_3$	3
FA 18:4	Fatty Acyls [FA]	277.222	277.21612	0.276	**[M+H]⁺**	$C_{18}H_{28}O_2$	3
FA 18:1	Fatty Acyls [FA]	283.301	283.26320	0.310	**[M+H]⁺**	$C_{18}H_{34}O_2$	3
FAHFA 35:4;O	Fatty Acyls [FA]	547.497	547.47223	0.446	**[M+H]⁺**	$C_{35}H_{62}O_4$	3
FA 20:1	Fatty Acyls [FA]	309.291	309.28021	0.545	**[M-H]⁻**	$C_{20}H_{38}O_2$	2
Citramalic acid	Fatty Acyls [FA]	147.045	147.02996	0.327	**[M-H]⁻**	$C_5H_8O_5$	3
FA 14:0	Fatty Acyls [FA]	227.209	227.20179	0.936	**[M-H]⁻**	$C_{14}H_{28}O_2$	2
FA 15:0	Fatty Acyls [FA]	241.231	241.21748	0.322	**[M-H]⁻**	$C_{15}H_{30}O_2$	2
CAR 3:1;O2	Fatty Acyls [FA]	246.112	246.09849	0.884	**[M-H]⁻**	$C_{11}H_{19}NO_6$	3
FA 16:1	Fatty Acyls [FA]	253.214	253.21752	1.827	**[M-H]⁻**	$C_{16}H_{30}O_2$	2
FA 16:0	Fatty Acyls [FA]	255.239	255.23311	0.061	**[M-H]⁻**	$C_{16}H_{32}O_2$	2
FA 14:1;O2	Fatty Acyls [FA]	257.181	257.17696	0.811	**[M-H]⁻**	$C_{14}H_{26}O_4$	3

FA 17:1	Fatty Acyls [FA]	267.231	267.23323	0.304	**$[M-H]^-$**	$C_{17}H_{32}O_2$	3
FA 17:0	Fatty Acyls [FA]	269.252	269.24873	1.001	**$[M-H]^-$**	$C_{17}H_{34}O_2$	2
FA 18:2	Fatty Acyls [FA]	279.243	279.23307	0.689	**$[M-H]^-$**	$C_{18}H_{32}O_2$	2
FA 18:1	Fatty Acyls [FA]	281.268	281.24875	0.683	**$[M-H]^-$**	$C_{18}H_{34}O_2$	2
FA 18:0	Fatty Acyls [FA]	283.269	283.26438	0.689	**$[M-H]^-$**	$C_{18}H_{36}O_2$	2
FA 20:5	Fatty Acyls [FA]	301.222	301.21744	0.358	**$[M-H]^-$**, $[M+Cl]^-$	$C_{20}H_{30}O_2$	3
FA 20:4	Fatty Acyls [FA]	303.222	303.23309	-0.105	**$[M-H]^-$**, $[M+Cl]^-$	$C_{20}H_{32}O_2$	2
FA 20:2	Fatty Acyls [FA]	307.265	307.26454	0.755	**$[M-H]^-$**	$C_{20}H_{36}O_2$	3
FA 22:6	Fatty Acyls [FA]	327.201	327.23336	2.769	**$[M-H]^-$**	$C_{22}H_{32}O_2$	2
FA 22:5	Fatty Acyls [FA]	329.252	329.24891	1.269	**$[M-H]^-$**	$C_{22}H_{34}O_2$	3
FA 22:4	Fatty Acyls [FA]	331.277	331.26455	0.175	**$[M-H]^-$**	$C_{22}H_{36}O_2$	2
FA 22:3	Fatty Acyls [FA]	333.202	333.27987	1.315	**$[M-H]^-$**	$C_{22}H_{38}O_2$	3
MG 22:6	Glycerolipids [GL]	403.322	403.28415	-0.327	**$[M+H]^+$**	$C_{25}H_{38}O_4$	3
DG 42:5	Glycerolipids [GL]	603.544	603.53473	0.114	**$[M+H]^+$**, $[M+Na]^+$	$C_{39}H_{70}O_4$	3
TG 50:4	Glycerolipids [GL]/Triradylglycerols	827.708	827.70934	-2.647	**$[M+H]^+$**, $[M+K]^+$, $[M+Na]^+$	$C_{53}H_{94}O_6$	2
TG 58:9	Glycerolipids [GL]/Triradylglycerols	929.763	929.75679	-2.622	**$[M+H]^+$**	$C_{61}H_{100}O_6$	2
TG 58:9	Glycerolipids [GL]/Triradylglycerols	933.813	933.78801	-2.511	**$[M+H]^+$**	$C_{61}H_{104}O_6$	3
TG 56:4	Glycerolipids [GL]/Triradylglycerols	911.829	911.80399	-2.441	**$[M+H]^+$**	$C_{59}H_{106}O_6$	2
TG 54:3	Glycerolipids [GL]/Triradylglycerols	801.662	801.69474	-2.264	**$[M+H]^+$**, $[M+K]^+$, $[M+Na]^+$	$C_{51}H_{92}O_6$	2
TG 52:4	Glycerolipids [GL]/Triradylglycerols	855.751	855.74149	-1.856	**$[M+H]^+$**, $[M+K]^+$, $[M+Na]^+$	$C_{55}H_{98}O_6$	2
TG 56:5	Glycerolipids [GL]/Triradylglycerols	909.795	909.78845	-1.794	**$[M+H]^+$**, $[M+Na]^+$	$C_{59}H_{104}O_6$	2
TG 50:3	Glycerolipids [GL]/Triradylglycerols	829.720	829.72353	-1.539	**$[M+H]^+$**	$C_{53}H_{96}O_6$	2
TG 52:4	Glycerolipids [GL]/Triradylglycerols	877.731	877.72748	-0.551	**$[M+H]^+$**	$C_{57}H_{96}O_6$	3
TG 46:2	Glycerolipids [GL]/Triradylglycerols	797.536	797.66288	-0.472	**$[M+Na]^+$**, $[M+K]^+$	$C_{49}H_{90}O_6$	2
TG 50:2	Glycerolipids [GL]/Triradylglycerols	853.741	853.72563	-0.084	**$[M+Na]^+$**, $[M+K]^+$	$C_{53}H_{98}O_6$	2
TG 56:3	Glycerolipids [GL]/Triradylglycerols	935.822	935.80391	0.206	**$[M+Na]^+$**, $[M+K]^+$	$C_{59}H_{108}O_6$	2
TG 52:5	Glycerolipids [GL]/Triradylglycerols	875.718	875.71025	0.444	**$[M+Na]^+$**, $[M+K]^+$	$C_{55}H_{96}O_6$	2
TG 53:2	Glycerolipids [GL]/Triradylglycerols	895.774	895.77292	0.638	**$[M+Na]^+$**, $[M+K]^+$	$C_{56}H_{104}O_6$	2
TG 52:2	Glycerolipids [GL]/Triradylglycerols	881.762	881.75713	1.124	**$[M+Na]^+$**, $[M+K]^+$	$C_{55}H_{102}O_6$	2
TG 54:5	Glycerolipids [GL]/Triradylglycerols	911.829	919.71581	1.178	**$[M+K]^+$**, $[M+Na]^+$	$C_{57}H_{100}O_6$	2
TG 54:3	Glycerolipids [GL]/Triradylglycerols	907.792	907.77289	1.274	**$[M+Na]^+$**, $[M+K]^+$	$C_{57}H_{104}O_6$	2
TG 46:1	Glycerolipids [GL]/Triradylglycerols	799.704	799.67915	1.554	**$[M+Na]^+$**, $[M+K]^+$	$C_{49}H_{92}O_6$	2
TG 52:3	Glycerolipids [GL]/Triradylglycerols	879.771	879.74158	1.654	**$[M+Na]^+$**, $[M+K]^+$, $[M+H]^+$	$C_{55}H_{100}O_6$	2

TG 51:2	Glycerolipids [GL]/Triradylglycerols	867.731	867.74179	1.894	**[M+Na]$^+$**, [M+K]$^+$	$C_{54}H_{100}O_6$	2
TG 54:4	Glycerolipids [GL]/Triradylglycerols	883.794	883.77561	0.171	**[M+H]$^+$**, [M+Na]$^+$	$C_{57}H_{102}O_6$	3
TG 48:2	Glycerolipids [GL]/Triradylglycerols	825.579	825.69452	0.314	**[M+Na]$^+$**	$C_{51}H_{94}O_6$	2
CPA 18:1	Glycerophospholipids [GP]	419.284	419.25573	0.062	**[M+H]$^+$**	$C_{21}H_{39}O_6P$	3
CL 36:4	Glycerophospholipids [GP]	911.828	925.52082	0.615	**[M+H]$^+$**, [M+K]$^+$, [M+Na]$^+$	$C_{45}H_{82}O_{15}P_2$	3
LPG 18:2	Glycerophospholipids [GP]	507.294	507.27355	1.394	**[M-H]$^-$**	$C_{24}H_{45}O_9P$	3
PA O-31:0	Glycerophospholipids [GP]/Glycerophosphates	621.411	621.48541	-3.591	**[M+H]$^+$**	$C_{34}H_{69}O_7P$	3
PA O-36:4	Glycerophospholipids [GP]/Glycerophosphates	683.472	683.49862	-3.488	**[M+H]$^+$**	$C_{39}H_{71}O_7P$	3
PA 42:10	Glycerophospholipids [GP]/Glycerophosphates	769.552	769.47829	-2.606	**[M+H]$^+$**	$C_{45}H_{69}O_8P$	3
PA 36:3	Glycerophospholipids [GP]/Glycerophosphates	721.572	721.47782	-0.064	**[M+Na]$^+$**, [M+K]$^+$	$C_{39}H_{71}O_8P$	3
PA O-40:6	Glycerophospholipids [GP]/Glycerophosphates	757.454	757.51427	-0.035	**[M+Na]$^+$**, [M+K]$^+$	$C_{43}H_{75}O_7P$	3
PA 38:4	Glycerophospholipids [GP]/Glycerophosphates	747.603	747.47255	-0.033	**[M+K]$^+$**, [M+H]$^+$, [M+Na]$^+$	$C_{41}H_{73}O_7P$	3
PA O-42:6	Glycerophospholipids [GP]/Glycerophosphates	785.585	785.54559	0.029	**[M+Na]$^+$**, [M+K]$^+$	$C_{45}H_{79}O_7P$	3
PA O-33:0	Glycerophospholipids [GP]/Glycerophosphates	649.454	649.51666	0.048	**[M+H]$^+$**	$C_{36}H_{73}O_7P$	3
PA 42:9	Glycerolipids [GL]	771.534	771.49362	-3.111	**[M+H]$^+$**	$C_{45}H_{71}O_8P$	3
PA 40:5	Glycerolipids [GL]	789.508	789.48313	-0.006	**[M+K]$^+$**, [M+Na]$^+$, [M+H]$^+$	$C_{43}H_{75}O_8P$	3
LPA 20:1	Glycerophospholipids [GP]/Glycerophosphates	487.291	487.27956	0.051	**[M+Na]$^+$, [M+K]$^+$**	$C_{23}H_{45}O_7P$	3
LPA O-20:4	Glycerophospholipids [GP]/Glycerophosphates	445.286	445.27138	0.052	**[M+H]$^+$**	$C_{23}H_{41}O_6P$	3
PA 32:1	Glycerophospholipids [GP]/Glycerophosphates	669.421	669.44662	0.053	**[M+Na]$^+$**, [M+K]$^+$	$C_{35}H_{67}O_8P$	3
PA 34:2	Glycerophospholipids [GP]/Glycerophosphates	695.487	695.46228	0.109	**[M+Na]$^+$**, [M+K]$^+$	$C_{37}H_{69}O_8P$	3
LPA 18:1	Glycerophospholipids [GP]/Glycerophosphates	459.307	459.24825	0.115	**[M+Na]$^+$**, [M+K]$^+$	$C_{21}H_{41}O_7P$	3
PA 38:3	Glycerophospholipids [GP]/Glycerophosphates	765.529	765.48318	0.201	**[M+K]$^+$**, [M+Na]$^+$	$C_{41}H_{75}O_8P$	3
LPA 18:2	Glycerophospholipids [GP]/Glycerophosphates	433.236	433.23645	1.282	**[M-H]$^-$**	$C_{21}H_{39}O_7P$	2
LPA 18:0	Glycerophospholipids [GP]/Glycerophosphates	437.268	437.26651	-1.941	**[M-H]$^-$**	$C_{21}H_{43}O_7P$	2
PA 36:3	Glycerophospholipids [GP]/Glycerophosphates	697.482	697.48139	0.045	**[M-H]$^-$**	$C_{39}H_{71}O_8P$	3
PA 38:4	Glycerophospholipids [GP]/Glycerophosphates	723.551	723.49641	-0.844	**[M-H]$^-$**, [M+Cl]$^-$	$C_{41}H_{73}O_8P$	3
PA 24:0	Glycerophospholipids [GP]/Glycerophosphates	644.395	644.42671	0.831	**[M+Na]$^+$**	$C_{32}H_{64}NO_8P$	3
PC 36:2	Glycerophospholipids [GP]/Glycerophosphocholines	786.651	786.59519	-4.899	**[M+H]$^+$**, [M+K]$^+$, [M+Na]$^+$	$C_{44}H_{84}NO_8P$	3
PC O-38:4	Glycerophospholipids [GP]/Glycerophosphocholines	796.648	796.61821	-4.435	**[M+H]$^+$**, [M+Na]$^+$, [M+K]$^+$	$C_{46}H_{86}NO_7P$	2
LPC 20:4	Glycerophospholipids [GP]/Glycerophosphocholines	544.362	544.33728	-4.349	**[[M+H]$^+$**, [M+Na]$^+$, [M+K]$^+$	$C_{28}H_{50}NO_7P$	3
PC O-36:5	Glycerophospholipids [GP]/Glycerophosphocholines	766.598	766.57285	-3.829	**[M+H]$^+$**	$C_{44}H_{80}NO_7P$	2
PC 36:6	Glycerophospholipids [GP]/Glycerophosphocholines	778.515	778.53554	-3.823	**[M+H]$^+$**	$C_{44}H_{76}NO_8P$	3
PC 36:3	Glycerophospholipids [GP]/Glycerophosphocholines	784.605	784.58088	-3.686	**[M+H]$^+$**, [M+Na]$^+$, [M+K]$^+$	$C_{44}H_{82}NO_8P$	2

PC O-36:5	Glycerophospholipids [GP]/Glycerophosphocholines	681.462	681.48301	-3.469	**[M+H]⁺**	$C_{39}H_{69}O_7P$	3
LPC 18:1	Glycerophospholipids [GP]/Glycerophosphocholines	522.374	522.35365	-3.357	**[M+H]⁺**, [M+K]⁺, [M+Na]⁺	$C_{26}H_{52}NO_7P$	2
PC 38:7	Glycerophospholipids [GP]/Glycerophosphocholines	804.546	804.55139	-3.124	**[M+H]⁺**	$C_{46}H_{78}NO_8P$	3
LPC 22:6	Glycerophospholipids [GP]/Glycerophosphocholines	801.491	801.46549	-2.866	**[M+Na]⁺**, [M+K]⁺	$C_{43}H_{71}O_{10}P$	3
PC O-40:9	Glycerophospholipids [GP]/Glycerophosphocholines	814.561	814.57262	-2.654	**[M+H]⁺**	$C_{48}H_{80}NO_7P$	3
PC 40:9	Glycerophospholipids [GP]/Glycerophosphocholines	828.566	828.55158	-2.626	**[M+H]⁺**	$C_{48}H_{78}NO_8P$	3
PC 38:3	Glycerophospholipids [GP]/Glycerophosphocholines	812.622	812.54135	-2.596	**[M+H]⁺**, [M+K]⁺, [M+Na]⁺	$C_{46}H_{86}NO_8P$	2
PC 40:8	Glycerophospholipids [GP]/Glycerophosphocholines	830.578	830.56729	-2.331	**[M+H]⁺**, [M+K]⁺, [M+Na]⁺	$C_{48}H_{80}NO_8P$	2
PC 42:9	Glycerophospholipids [GP]/Glycerophosphocholines	856.579	856.58310	-2.067	**[M+H]⁺**	$C_{50}H_{82}NO_8P$	3
PC O-30:0	Glycerophospholipids [GP]/Glycerophosphocholines	703.561	703.52583	-1.534	**[M+H]⁺**	$C_{39}H_{75}O_8P$	3
PC 36:4	Glycerophospholipids [GP]/Glycerophosphocholines	782.597	782.57031	-1.324	**[M+H]⁺**	$C_{44}H_{80}NO_8P$	2
PC 34:0	Glycerophospholipids [GP]/Glycerophosphocholines	762.601	762.60065	-0.685	**[M+H]⁺**	$C_{42}H_{84}NO_8P$	2
PC O-34:1	Glycerophospholipids [GP]/Glycerophosphocholines	746.686	746.60573	-0.614	**[M+H]⁺**, [M+K]⁺, [M+Na]⁺	$C_{42}H_{84}NO_7P$	2
PC 32:2	Glycerophospholipids [GP]/Glycerophosphocholines	730.535	730.53785	-0.508	**[M+H]⁺**	$C_{40}H_{76}NO_8P$	2
PC 32:1	Glycerophospholipids [GP]/Glycerophosphocholines	770.558	770.50966	-0.402	**[M+K]⁺**, [M+Na]⁺, [M+H]⁺	$C_{40}H_{78}NO_8P$	2
PC 32:0	Glycerophospholipids [GP]/Glycerophosphocholines	772.599	772.52522	-0.382	**[M+K]⁺**, [M+Na]⁺, [M+H]⁺	$C_{40}H_{80}NO_8P$	2
PC 38:5	Glycerophospholipids [GP]/Glycerophosphocholines	808.599	808.58554	-0.356	**[M+H]⁺**	$C_{46}H_{82}NO_8P$	2
PC O-36:2	Glycerophospholipids [GP]/Glycerophosphocholines	772.599	772.62126	-0.341	**[M+H]⁺**, [M+K]⁺	$C_{44}H_{86}NO_7P$	2
PC O-31:1	Glycerophospholipids [GP]/Glycerophosphocholines	706.526	706.50151	-0.332	**[M+H]⁺**, [M+K]⁺	$C_{37}H_{72}NO_9P$	3
PC O-42:4	Glycerophospholipids [GP]/Glycerophosphocholines	874.682	874.66576	-0.275	**[M+Na]⁺**, [M+H]⁺, [M+K]⁺	$C_{50}H_{94}NO_7P$	3
LPC 16:0	Glycerophospholipids [GP]/Glycerophosphocholines	496.343	496.33977	-0.253	**[M+H]⁺**, [M+K]⁺, [M+Na]⁺	$C_{24}H_{50}NO_7P$	2
LPC 18:0	Glycerophospholipids [GP]/Glycerophosphocholines	524.401	524.37106	-0.235	**[M+H]⁺**, [M+Na]⁺, [M+K]⁺	$C_{26}H_{54}NO_7P$	2
PC 20:1	Glycerophospholipids [GP]/Glycerophosphocholines	564.291	564.36595	-0.198	**[M+H]⁺**, [M+K]⁺, [M+Na]⁺	$C_{28}H_{54}NO_8P$	3
LPC 20:2	Glycerophospholipids [GP]/Glycerophosphocholines	548.398	548.37105	-0.197	**[M+H]⁺**, [M+Na]⁺	$C_{28}H_{54}NO_7P$	3
LPC 18:1	Glycerophospholipids [GP]/Glycerophosphocholines	592.423	592.39726	-0.187	**[M+H]⁺**, [M+K]⁺, [M+Na]⁺	$C_{30}H_{58}NO_8P$	3
PC 38:6	Glycerophospholipids [GP]/Glycerophosphocholines	806.588	806.57025	-0.085	**[M+H]⁺**	$C_{46}H_{80}NO_8P$	2
PC O-30:0	Glycerophospholipids [GP]/Glycerophosphocholines	692.553	692.55883	-0.049	**[M+H]⁺**, [M+Na]⁺, [M+K]⁺	$C_{38}H_{78}NO_7P$	3
PC 30:0	Glycerophospholipids [GP]/Glycerophosphocholines	744.497	744.49407	-0.046	**[M+K]⁺**, [M+Na]⁺, [M+H]⁺	$C_{38}H_{76}NO_8P$	2
PC O-38:4	Glycerophospholipids [GP]/Glycerophosphocholines	763.492	763.46764	-0.003	**[M+K]⁺**, [M+H]⁺, [M+Na]⁺	$C_{41}H_{73}O_8P$	3
PC O-32:2	Glycerophospholipids [GP]/Glycerophosphocholines	551.511	551.50341	0.007	**[M+H]⁺**, [M+Na]⁺	$C_{35}H_{66}O_4$	3
PC dO-34:4	Glycerophospholipids [GP]/Glycerophosphocholines	748.579	748.56149	0.019	**[M+Na]⁺**, [M+K]⁺	$C_{42}H_{80}NO_6P$	3
LPC O-16:2	Glycerophospholipids [GP]/Glycerophosphocholines	478.322	478.32923	0.029	**[M+H]⁺**, [M+K]⁺, [M+Na]⁺	$C_{24}H_{48}NO_6P$	3
LPC O-14:1	Glycerophospholipids [GP]/Glycerophosphocholines	450.299	450.29915	0.035	**[M-H]⁻**	$C_{22}H_{46}NO_6P$	3

LPC O-18:2	Glycerophospholipids [GP]/Glycerophosphocholines	506.371	506.36053	0.046	**$[M+H]^+$**, $[M+Na]^+$, $[M+K]^+$	$C_{26}H_{52}NO_6P$	3
PC 21:1;O	Glycerophospholipids [GP]/Glycerophosphocholines	594.374	594.37660	0.046	**$[M+H]^+$**, $[M+Na]^+$, $[M+K]^+$	$C_{29}H_{56}NO_9P$	3
PC 40:6	Glycerophospholipids [GP]/Glycerophosphocholines	834.601	834.60136	0.049	**$[M+H]^+$**	$C_{48}H_{84}NO_8P$	2
LPC O-16:1	Glycerophospholipids [GP]/Glycerophosphocholines	480.369	480.34488	0.057	**$[M+H]^+$**	$C_{24}H_{50}NO_6P$	2
PC 34:3	Glycerophospholipids [GP]/Glycerophosphocholines	756.566	756.55419	0.063	**$[M+H]^+$**	$C_{42}H_{78}NO_8P$	2
PC 21:1	Glycerophospholipids [GP]/Glycerophosphocholines	578.412	578.38163	0.064	**$[M+H]^+$**, $[M+Na]^+$, $[M+K]^+$	$C_{29}H_{56}NO_8P$	3
PC 33:0	Glycerophospholipids [GP]/Glycerophosphocholines	786.591	786.54110	0.064	**$[M+K]^+$**, $[M+Na]^+$, $[M+H]^+$	$C_{41}H_{82}NO_8P$	2
PC 26:1	Glycerophospholipids [GP]/Glycerophosphocholines	648.485	648.45987	0.065	**$[M+H]^+$**, $[M+K]^+$, $[M+Na]^+$	$C_{34}H_{66}NO_8P$	3
LPC O-16:0	Glycerophospholipids [GP]/Glycerophosphocholines	482.411	482.36052	0.071	**$[M+H]^+$**	$C_{24}H_{52}NO_6P$	3
PC 26:0	Glycerophospholipids [GP]/Glycerophosphocholines	688.412	688.43153	0.075	**$[M+K]^+$**, $[M+H]^+$	$C_{34}H_{68}NO_8P$	3
PC O-32:0	Glycerophospholipids [GP]/Glycerophosphocholines	720.596	720.59017	0.076	**$[M+H]^+$**, $[M+K]^+$, $[M+Na]^+$	$C_{40}H_{82}NO_7P$	3
PC O-44:4	Glycerophospholipids [GP]/Glycerophosphocholines	900.744	900.68175	0.079	**$[M+Na]^+$**, $[M+K]^+$, $[M+H]^+$	$C_{52}H_{96}NO_7P$	2
LPC O-18:1	Glycerophospholipids [GP]/Glycerophosphocholines	508.382	508.37618	0.081	**$[M+H]^+$**, $[M+K]^+$, $[M+Na]^+$	$C_{26}H_{54}NO_6P$	3
LPC 22:6	Glycerophospholipids [GP]/Glycerophosphocholines	568.371	568.33958	0.084	**$[M+H]^+$**	$C_{30}H_{50}NO_7P$	3
PC O-36:1	Glycerophospholipids [GP]/Glycerophosphocholines	774.670	774.63723	0.089	**$[M+H]^+$**	$C_{44}H_{88}NO_7P$	3
PC O-32:1	Glycerophospholipids [GP]/Glycerophosphocholines	740.581	740.55654	0.091	**$[M+Na]^+$**, $[M+H]^+$	$C_{40}H_{80}NO_7P$	2
LPC 22:6	Glycerophospholipids [GP]/Glycerophosphocholines	526.322	526.29278	0.092	**$[M+H]^+$**	$C_{27}H_{44}NO_7P$	3
PC O-42:5	Glycerophospholipids [GP]/Glycerophosphocholines	872.731	872.65043	0.104	**$[M+Na]^+$**, $[M+K]^+$, $[M+H]^+$	$C_{50}H_{92}NO_7P$	3
LPC O-20:0	Glycerophospholipids [GP]/Glycerophosphocholines	538.451	538.42316	0.108	**$[M+H]^+$**	$C_{28}H_{60}NO_6P$	3
PC 24:0	Glycerophospholipids [GP]/Glycerophosphocholines	622.469	622.44425	0.112	**$[M+H]^+$**, $[M+K]^+$, $[M+Na]^+$	$C_{32}H_{64}NO_8P$	3
LPC 18:2	Glycerophospholipids [GP]/Glycerophosphocholines	520.333	520.33979	0.118	**$[M+H]^+$**	$C_{26}H_{50}NO_7P$	2
PC dO-36:4	Glycerophospholipids [GP]/Glycerophosphocholines	776.622	776.59293	0.119	**$[M+Na]^+$**, $[M+K]^+$	$C_{44}H_{84}NO_6P$	3
LPC 15:0	Glycerophospholipids [GP]/Glycerophosphocholines	480.328	480.31018	0.139	**$[M-H]^-$**	$C_{23}H_{48}NO_7P$	3
PC 40:4	Glycerophospholipids [GP]/Glycerophosphocholines	846.551	846.63481	0.144	**$[M+Na]^+$**, $[M+H]^+$, $[M+K]^+$	$C_{48}H_{90}NO_7P$	3
PC O-38:1	Glycerophospholipids [GP]/Glycerophosphocholines	840.649	840.62446	0.151	**$[M+K]^+$**, $[M+H]^+$	$C_{46}H_{92}NO_7P$	3
PC O-38:5	Glycerophospholipids [GP]/Glycerophosphocholines	832.561	832.56186	0.153	**$[M+K]^+$**, $[M+Na]^+$, $[M+H]^+$	$C_{46}H_{84}NO_7P$	2
PC 21:1;O2	Glycerophospholipids [GP]/Glycerophosphocholines	610.318	610.37153	0.156	**$[M+H]^+$**, $[M+K]^+$, $[M+Na]^+$	$C_{29}H_{56}NO_{10}P$	3
PC 44:0	Glycerophospholipids [GP]/Glycerophosphocholines	817.473	817.66815	0.165	**$[M+H]^+$**	$C_{47}H_{93}O_8P$	3
PC 25:1;O	Glycerophospholipids [GP]/Glycerophosphocholines	650.459	650.43921	0.183	**$[M+H]^+$**, $[M+K]^+$, $[M+Na]^+$	$C_{33}H_{64}NO_9P$	3
PC 19:1	Glycerophospholipids [GP]/Glycerophosphocholines	550.401	550.35036	0.199	**$[M+H]^+$**, $[M+Na]^+$, $[M+K]^+$	$C_{27}H_{52}NO_8P$	3
PC O-40:5	Glycerophospholipids [GP]/Glycerophosphocholines	860.602	860.59319	0.203	**$[M+K]^+$**, $[M+H]^+$, $[M+Na]^+$	$C_{48}H_{88}NO_7P$	3
PC O-38:2	Glycerophospholipids [GP]/Glycerophosphocholines	800.671	800.65301	0.229	**$[M+H]^+$**	$C_{46}H_{90}NO_7P$	3
LPC 20:1	Glycerophospholipids [GP]/Glycerophosphocholines	550.401	550.38675	0.238	**$[M+H]^+$**, $[M+K]^+$, $[M+Na]^+$	$C_{28}H_{56}NO_7P$	3

PC 38:1	Glycerophospholipids [GP]/Glycerophosphocholines	854.745	854.69993	0.249	**$[M+H]^+$**	$C_{50}H_{96}NO_7P$	2
PC O-42:6	Glycerophospholipids [GP]/Glycerophosphocholines	870.655	870.63493	0.292	**$[M+Na]^+$**, $[M+K]^+$, $[M+H]^+$	$C_{50}H_{90}NO_7P$	3
PC O-38:3	Glycerophospholipids [GP]/Glycerophosphocholines	798.662	798.63739	0.327	**$[M+H]^+$**	$C_{46}H_{88}NO_7P$	3
PC O-34:2	Glycerophospholipids [GP]/Glycerophosphocholines	744.615	744.59023	0.328	**$[M+H]^+$**, $[M+K]^+$	$C_{42}H_{82}NO_7P$	2
PC 34:1	Glycerophospholipids [GP]/Glycerophosphocholines	798.542	798.54104	0.339	**$[M+K]^+$**, $[M+Na]^+$, $[M+H]^+$	$C_{42}H_{82}NO_8P$	2
LPC O-18:1	Glycerophospholipids [GP]/Glycerophosphocholines	464.315	464.31516	0.343	**$[M-H]^-$**	$C_{23}H_{48}NO_6P$	2
LPC 20:3	Glycerophospholipids [GP]/Glycerophosphocholines	546.381	546.35556	0.351	**$[M+H]^+$**	$C_{28}H_{52}NO_7P$	3
PC O-36:4	Glycerophospholipids [GP]/Glycerophosphocholines	768.665	768.59072	0.459	**$[M+H]^+$**	$C_{44}H_{82}NO_7P$	2
PC 38:1	Glycerophospholipids [GP]/Glycerophosphocholines	816.662	816.64806	0.536	**$[M+H]^+$**, $[M+K]^+$	$C_{46}H_{90}NO_8P$	3
PC O-40:6	Glycerophospholipids [GP]/Glycerophosphocholines	820.641	820.62198	0.671	**$[M+H]^+$**, $[M+Na]^+$	$C_{48}H_{86}NO_7P$	3
PC O-36:3	Glycerophospholipids [GP]/Glycerophosphocholines	770.558	770.60615	0.779	**$[M+H]^+$**	$C_{44}H_{84}NO_7P$	2
PC O-38:6	Glycerophospholipids [GP]/Glycerophosphocholines	792.614	792.58907	0.942	**$[M+H]^+$**, $[M+K]^+$	$C_{46}H_{82}NO_7P$	2
PC 40:4	Glycerophospholipids [GP]/Glycerophosphocholines	838.657	838.63281	0.985	**$[M+H]^+$**, $[M+Na]^+$, $[M+K]^+$	$C_{48}H_{88}NO_8P$	2
PC 38:2	Glycerophospholipids [GP]/Glycerophosphocholines	814.671	814.63243	1.516	**$[M+H]^+$**, $[M+K]^+$	$C_{46}H_{88}NO_8P$	2
LPC 14:1	Glycerophospholipids [GP]/Glycerophosphocholines	464.279	464.27852	0.553	**$[M-H]^-$**	$C_{22}H_{44}NO_7P$	3
LPC 14:0	Glycerophospholipids [GP]/Glycerophosphocholines	466.293	466.29401	-0.208	**$[M-H]^-$**	$C_{22}H_{46}NO_7P$	3
PC 16:0	Glycerophospholipids [GP]/Glycerophosphocholines	508.336	508.30512	1.263	**$[M-H]^-$**	$C_{24}H_{48}NO_8P$	3
PC 34:2	Glycerophospholipids [GP]/Glycerophosphocholines	758.574	758.56961	0.235	**$[M+H]^+$**	$C_{42}H_{80}NO_8P$	2
PC dO-37:4	Glycerophospholipids [GP]/Glycerophosphocholines	766.625	766.61071	-0.251	**$[M-H]^-$**	$C_{45}H_{86}NO_6P$	3
PC 36:1	Glycerophospholipids [GP]/Glycerophosphocholines	788.632	788.61614	-0.306	**$[M+H]^+$**, $[M+K]^+$	$C_{44}H_{86}NO_8P$	2
PC 22:1	Glycerophospholipids [GP]/Glycerophosphocholines	614.223	614.37953	0.496	**$[M+Na]^+$**	$C_{30}H_{58}NO_8P$	3
PE O-30:0	Glycerophospholipids [GP]/Glycerophosphoethanolamines	650.519	650.50957	-3.552	**$[M+H]^+$**	$C_{35}H_{72}NO_7P$	3
PE O-38:6	Glycerophospholipids [GP]/Glycerophosphoethanolamines	706.526	707.49862	-3.365	**$[M+H]^+$**, $[M+Na]^+$	$C_{41}H_{71}O_7P$	3
PE O-42:7	Glycerophospholipids [GP]/Glycerophosphoethanolamines	804.635	804.58778	-2.992	**$[M+H]^+$**	$C_{47}H_{82}NO_7P$	3
PE 42:7	Glycerophospholipids [GP]/Glycerophosphoethanolamines	818.479	818.56699	-2.834	**$[M+H]^+$**	$C_{47}H_{80}NO_8P$	3
LPE 20:4	Glycerophospholipids [GP]/Glycerophosphoethanolamines	540.272	540.24859	-0.299	**$[M+K]^+$**, $[M+H]^+$, $[M+Na]^+$	$C_{25}H_{44}NO_7P$	3
PE 29:2	Glycerophospholipids [GP]/Glycerophosphoethanolamines	646.469	646.44411	-0.183	**$[M+H]^+$**	$C_{34}H_{64}NO_8P$	3
PE 36:1	Glycerophospholipids [GP]/Glycerophosphoethanolamines	746.597	746.56931	-0.139	**$[M+H]^+$**, $[M+Na]^+$	$C_{41}H_{80}NO_8P$	2
PE 30:0	Glycerophospholipids [GP]/Glycerophosphoethanolamines	664.511	664.49102	-0.131	**$[M+H]^+$**	$C_{35}H_{70}NO_8P$	3
PE 42:9	Glycerophospholipids [GP]/Glycerophosphoethanolamines	814.561	814.53655	-1.793	**$[M+H]^+$**	$C_{47}H_{76}NO_8P$	3
PE O-36:0	Glycerophospholipids [GP]/Glycerophosphoethanolamines	734.676	734.60574	-0.101	**$[M+H]^+$**	$C_{41}H_{84}NO_7P$	3
LPE 18:0	Glycerophospholipids [GP]/Glycerophosphoethanolamines	482.351	482.32414	-0.002	**$[M+H]^+$**, $[M+K]^+$	$C_{23}H_{48}NO_7P$	2
LPE 18:1	Glycerophospholipids [GP]/Glycerophosphoethanolamines	480.312	480.30849	0.012	**$[M+H]^+$**	$C_{23}H_{46}NO_7P$	2

LPE 20:0	Glycerophospholipids [GP]/Glycerophosphoethanolamines	510.381	510.35541	0.074	**$[M+H]^+$**	$C_{25}H_{52}NO_7P$	2
PE 28:0	Glycerophospholipids [GP]/Glycerophosphoethanolamines	636.438	636.45997	0.083	**$[M+H]^+$**, $[M+K]^+$, $[M+Na]^+$	$C_{33}H_{66}NO_8P$	3
PE 36:2	Glycerophospholipids [GP]/Glycerophosphoethanolamines	744.586	744.55392	0.104	**$[M+H]^+$**, $[M+Na]^+$	$C_{41}H_{78}NO_8P$	2
PE 30:1	Glycerophospholipids [GP]/Glycerophosphoethanolamines	662.442	662.47561	0.105	**$[M+H]^+$**	$C_{35}H_{68}NO_8P$	3
PE 20:0	Glycerophospholipids [GP]/Glycerophosphoethanolamines	562.311	562.29059	0.115	**$[M+K]^+$**, $[M+H]^+$, $[M+Na]^+$	$C_{25}H_{50}NO_8P$	3
PE 27:1	Glycerophospholipids [GP]/Glycerophosphoethanolamines	606.413	620.42859	0.116	**$[M+H]^+$**, $[M+K]^+$, $[M+Na]^+$	$C_{32}H_{62}NO_8P$	3
PE O-42:5	Glycerophospholipids [GP]/Glycerophosphoethanolamines	808.688	808.62083	0.134	**$[M+H]^+$**	$C_{47}H_{86}NO_7P$	3
PE 28:2	Glycerophospholipids [GP]/Glycerophosphoethanolamines	632.445	632.42869	0.169	**$[M+H]^+$**	$C_{33}H_{62}NO_8P$	3
PE 28:1	Glycerophospholipids [GP]/Glycerophosphoethanolamines	606.413	606.40299	0.196	**$[M+H]^+$**, $[M+Na]^+$	$C_{31}H_{60}NO_8P$	3
PE 42:4	Glycerophospholipids [GP]/Glycerophosphoethanolamines	824.632	824.61657	0.205	**$[M+H]^+$**	$C_{47}H_{86}NO_8P$	3
PE 38:1	Glycerophospholipids [GP]/Glycerophosphoethanolamines	774.611	774.60082	0.276	**$[M+H]^+$**, $[M+Na]^+$, $[M+K]^+$	$C_{43}H_{84}NO_8P$	2
PE 34:0	Glycerophospholipids [GP]/Glycerophosphoethanolamines	718.541	718.53932	0.292	**$[M-H]^-$**, $[M+Cl]^-$	$C_{39}H_{78}NO_8P$	2
PE 40:3	Glycerophospholipids [GP]/Glycerophosphoethanolamines	798.632	798.60109	0.298	**$[M+H]^+$**	$C_{45}H_{84}NO_8P$	3
PE 38:4	Glycerophospholipids [GP]/Glycerophosphoethanolamines	768.576	768.55409	0.419	**$[M+H]^+$**, $[M+Na]^+$	$C_{43}H_{78}NO_8P$	2
PE 12:0	Glycerophospholipids [GP]/Glycerophosphoethanolamines	412.214	412.20957	0.425	**$[M+H]^+$**	$C_{17}H_{34}NO_8P$	3
LPE 18:2	Glycerophospholipids [GP]/Glycerophosphoethanolamines	478.322	478.29299	0.449	**$[M+H]^+$**, $[M+Na]^+$	$C_{23}H_{44}NO_7P$	3
LPE 20:4	Glycerophospholipids [GP]/Glycerophosphoethanolamines	500.279	500.27906	0.594	**$[M-H]^-$**	$C_{25}H_{44}NO_7P$	3
PE 40:1	Glycerophospholipids [GP]/Glycerophosphoethanolamines	802.505	802.63212	0.712	**$[M+H]^+$**	$C_{45}H_{88}NO_8P$	3
PE 42:8	Glycerophospholipids [GP]/Glycerophosphoethanolamines	838.561	838.53671	1.161	**$[M+Na]^+$**, $[M+H]^+$	$C_{47}H_{78}NO_8P$	3
PE 40:2	Glycerophospholipids [GP]/Glycerophosphoethanolamines	800.553	800.61639	1.393	**$[M+H]^+$**	$C_{45}H_{86}NO_8P$	2
LPE 22:4	Glycerophospholipids [GP]/Glycerophosphoethanolamines	528.334	528.30999	0.808	**$[M-H]^-$**	$C_{27}H_{48}NO_7P$	3
PE O-34:1	Glycerophospholipids [GP]/Glycerophosphoethanolamines	702.554	702.54427	-0.063	**$[M-H]^-$**	$C_{39}H_{78}NO_7P$	3
PC 33:3	Glycerophospholipids [GP]/Glycerophosphoethanolamines	742.539	742.53825	0.161	**$[M+H]^+$**	$C_{41}H_{76}NO_8P$	2
PE O-38:6	Glycerophospholipids [GP]/Glycerophosphoethanolamines	750.535	750.54368	0.042	**$[M+H]^+$**, $[M+Na]^+$	$C_{43}H_{76}NO_7P$	2
PG 39:8	Glycerophospholipids [GP]/Glycerophosphoglycerols	805.552	805.49831	-3.869	**$[M+H]^+$**	$C_{45}H_{73}O_{10}P$	3
PG 38:5	Glycerophospholipids [GP]/Glycerophosphoglycerols	835.572	835.48881	0.259	**$[M+K]^+$**	$C_{44}H_{77}O_{10}P$	3
PI 40:7	Glycerophospholipids [GP]/Glycerophosphoinositols	909.559	909.54688	-1.812	**$[M+H]^+$**, $[M+Na]^+$	$C_{49}H_{81}O_{13}P$	3
LPI 22:6	Glycerophospholipids [GP]/Glycerophosphoinositols	645.328	645.30369	0.379	**$[M+H]^+$**	$C_{31}H_{49}O_{12}P$	3
PI-Cer 36:1	Glycerophospholipids [GP]/Glycerophosphoinositols	808.599	808.56945	0.399	**$[M+H]^+$**, $[M+K]^+$, $[M+Na]^+$	$C_{42}H_{82}NO_{11}P$	3
LPI 18:2	Glycerophospholipids [GP]/Glycerophosphoinositols	597.331	597.30428	1.094	**$[M+H]^+$**	$C_{27}H_{49}O_{12}P$	3
LPI 18:0	Glycerophospholipids [GP]/Glycerophosphoinositols	599.361	599.31944	1.246	**$[M-H]^-$**	$C_{27}H_{53}O_{12}P$	2
PS O-29:1	Glycerophospholipids [GP]/Glycerophosphoserines	678.472	678.46714	-4.873	**$[M+H]^+$**, $[M+Na]^+$, $[M+K]^+$	$C_{35}H_{68}NO_9P$	3
PS O-35:0	Glycerophospholipids [GP]/Glycerophosphoserines	764.553	764.57765	-3.022	**$[M+H]^+$**	$C_{41}H_{82}NO_9P$	3

PS O-39:2	Glycerophospholipids [GP]/Glycerophosphoserines	816.662	816.60901	-2.827	**[M+H]+**	$C_{45}H_{86}NO_9P$	3
PS 32:1	Glycerophospholipids [GP]/Glycerophosphoserines	734.558	734.49639	-0.369	**[M+H]+**, [M+K]+	$C_{38}H_{72}NO_{10}P$	3
PS 28:1	Glycerophospholipids [GP]/Glycerophosphoserines	678.472	678.43389	-0.353	**[M+H]+**, [M+K]+	$C_{34}H_{64}NO_{10}P$	3
PS 31:2	Glycerophospholipids [GP]/Glycerophosphoserines	718.496	718.46512	-0.331	**[M+H]+**	$C_{37}H_{68}NO_{10}P$	3
PS 32:3	Glycerophospholipids [GP]/Glycerophosphoserines	730.490	730.46505	-0.322	**[M+H]+**, [M+K]+, [M+Na]+	$C_{38}H_{68}NO_{10}P$	3
PS 29:0	Glycerophospholipids [GP]/Glycerophosphoserines	666.459	666.43402	-0.314	**[M+H]+**, [M+Na]+, [M+K]+	$C_{33}H_{64}NO_{10}P$	3
PS O-32:2	Glycerophospholipids [GP]/Glycerophosphoserines	718.525	718.50159	-0.309	**[M+H]+**, [M+K]+, [M+Na]+	$C_{38}H_{72}NO_9P$	3
PS 30:1	Glycerophospholipids [GP]/Glycerophosphoserines	706.526	706.46516	-0.309	**[M+H]+**, [M+K]+, [M+Na]+	$C_{36}H_{68}NO_{10}P$	3
PS O-28:1	Glycerophospholipids [GP]/Glycerophosphoserines	664.451	664.45464	-0.269	**[M+H]+**, [M+Na]+, [M+K]+	$C_{34}H_{66}NO_9P$	3
PS 27:0	Glycerophospholipids [GP]/Glycerophosphoserines	690.458	690.43383	-0.248	**[M+H]+**, [M+K]+	$C_{35}H_{64}NO_{10}P$	3
PS O-31:2	Glycerophospholipids [GP]/Glycerophosphoserines	704.563	704.48606	0.059	**[M+H]+**, [M+K]+	$C_{37}H_{70}NO_9P$	3
PS O-36:3	Glycerophospholipids [GP]/Glycerophosphoserines	810.544	810.50464	0.075	**[M+K]+**, [M+H]+	$C_{42}H_{78}NO_9P$	3
PS 29:0	Glycerophospholipids [GP]/Glycerophosphoserines	694.490	694.46537	0.129	**[M+H]+**, [M+Na]+, [M+K]+	$C_{35}H_{68}NO_{10}P$	3
PS O-30:2	Glycerophospholipids [GP]/Glycerophosphoserines	712.461	712.45249	0.132	**[M+Na]+**, [M+K]+, [M+H]+	$C_{36}H_{68}NO_9P$	3
PS 29:1	Glycerophospholipids [GP]/Glycerophosphoserines	692.474	692.44969	0.159	**[M+H]+**, [M+K]+, [M+Na]+	$C_{35}H_{66}NO_{10}P$	3
PS 25:0	Glycerophospholipids [GP]/Glycerophosphoserines	638.427	638.40287	0.189	**[M+H]+**, [M+Na]+, [M+K]+	$C_{31}H_{60}NO_{10}P$	3
PS O-38:3	Glycerophospholipids [GP]/Glycerophosphoserines	800.553	800.58052	0.504	**[M+H]+**	$C_{44}H_{82}NO_9P$	3
PS O-30:1	Glycerophospholipids [GP]/Glycerophosphoserines	714.495	714.47019	2.818	**[M+Na]+**, [M+H]+	$C_{36}H_{70}NO_9P$	3
PS O-37:2	Glycerophospholipids [GP]/Glycerophosphoserines	826.591	826.53947	3.728	**[M+K]+**, [M+H]+	$C_{43}H_{82}NO_9P$	3
PS 36:1	Glycerophospholipids [GP]/Glycerophosphoserines	524.334	524.29997	1.102	**[M-H]-**	$C_{24}H_{48}NO_9P$	3
PS 25:0	Glycerophospholipids [GP]/Glycerophosphoserines	660.424	660.38482	0.176	**[M+Na]+**	$C_{31}H_{60}NO_{10}P$	3
LPS 22:1	Glycerophospholipids [GP]/Glycerophosphoserines	602.361	602.34291	0.116	**[M+Na]+**	$C_{28}H_{54}NO_9P$	3
Hex2Cer 42:2;O2	Sphingolipids [SP]	1010.714	1010.68933	-0.603	**[M+K]+**, [M+Na]+	$C_{54}H_{101}NO_{13}$	3
HexCer 42:2;O2	Sphingolipids [SP]	832.678	832.66371	-0.343	**[M+Na]+**, [M+K]+	$C_{48}H_{91}NO_8$	2
EPC 32:1;O2	Sphingolipids [SP]	703.561	703.53827	-0.333	**[M+H]+**, [M+Na]+	$C_{38}H_{75}N_2O_7P$	3
Cer 42:2;O	Sphingolipids [SP]	632.658	632.63397	-0.21	**[M+H]+**, [M+Na]+	$C_{42}H_{81}NO_2$	3
Hex2Cer 34:1;O2	Sphingolipids [SP]	900.611	900.58074	-0.198	**[M+K]+**, [M+Na]+	$C_{46}H_{87}NO_{13}$	2
EPC 37:1;O3	Sphingolipids [SP]	757.542	757.52555	-0.071	**[M+K]+**, [M+H]+, [M+Na]+	$C_{39}H_{79}N_2O_7P$	2
Cer 34:1;O	Sphingolipids [SP]	522.552	522.52436	0.012	**[M+H]+**	$C_{34}H_{67}NO_2$	3
CerP 44:2;O2	Sphingolipids [SP]	778.634	778.60839	0.027	**[M+Na]+**, [M+K]+	$C_{44}H_{86}NO_6P$	3
CerP 34:0	Sphingolipids [SP]	620.523	620.50138	0.029	**[M+H]+**	$C_{34}H_{70}NO_6P$	3
CerP 32:1	Sphingolipids [SP]	590.528	590.45448	0.048	**[M+H]+**	$C_{32}H_{64}NO_6P$	3
CerP 28:0	Sphingolipids [SP]	536.433	536.40749	0.124	**[M+H]+**	$C_{28}H_{58}NO_6P$	3

CerP 36:1;O2	Sphingolipids [SP]	668.504	668.49891	0.135	**[M+Na]$^+$**, [M+K]$^+$	$C_{36}H_{72}NO_6P$	3
Cer 32:0	Sphingolipids [SP]	512.531	512.50378	0.149	**[M+H]$^+$**	$C_{32}H_{65}NO_3$	3
CerP 34:1;O2	Sphingolipids [SP]	616.471	616.47116	0.018	**[M-H]$^-$**	$C_{34}H_{68}NO_6P$	2
SHexCer 36:1;O3	Sphingolipids [SP]/Acidic glycosphingolipids	824.632	824.55221	-3.507	**[M+H]$^+$**, [M+Na]$^+$	$C_{42}H_{81}NO_{12}S$	3
SM 40:1	Sphingolipids [SP]/Phosphosphingolipids	787.690	787.66547	-4.322	**[M+H]$^+$**, [M+Na]$^+$, [M+K]$^+$	$C_{45}H_{91}N_2O_6P$	2
SM 38:0	Sphingolipids [SP]/Phosphosphingolipids	761.595	761.65266	-0.477	**[M+H]$^+$**	$C_{43}H_{89}N_2O_6P$	3
SM 35:1;O2	Sphingolipids [SP]/Phosphosphingolipids	717.615	717.59022	-0.445	**[M+H]$^+$**, [M+Na]$^+$	$C_{40}H_{81}N_2O_6P$	2
SM 36:2;O2	Sphingolipids [SP]/Phosphosphingolipids	767.571	767.54608	-0.305	**[M+K]$^+$**, [M+H]$^+$, [M+Na]$^+$	$C_{41}H_{81}N_2O_6P$	2
SM 34:1	Sphingolipids [SP]/Phosphosphingolipids	725.581	725.55669	-0.139	**[M+Na]$^+$**, [M+K]$^+$, [M+H]$^+$	$C_{39}H_{79}N_2O_6P$	2
SM 37:1;O2	Sphingolipids [SP]/Phosphosphingolipids	745.621	745.62176	-0.024	**[M+H]$^+$**, [M+Na]$^+$	$C_{42}H_{85}N_2O_6P$	2
SM 38:2;O2	Sphingolipids [SP]/Phosphosphingolipids	795.583	795.57769	0.059	**[M+K]$^+$**, [M+H]$^+$	$C_{43}H_{85}N_2O_6P$	2
SM 32:1;O2	Sphingolipids [SP]/Phosphosphingolipids	697.525	697.52549	0.073	**[M+Na]$^+$**, [M+H]$^+$, [M+K]$^+$	$C_{37}H_{75}N_2O_6P$	2
SM 34:2	Sphingolipids [SP]/Phosphosphingolipids	723.566	723.54119	0.121	**[M+Na]$^+$**, [M+H]$^+$, [M+K]$^+$	$C_{39}H_{77}N_2O_6P$	2
SM 34:0	Sphingolipids [SP]/Phosphosphingolipids	727.547	727.57251	0.122	**[M+Na]$^+$**, [M+H]$^+$, [M+K]$^+$	$C_{39}H_{81}N_2O_6P$	2
SM 42:2	Sphingolipids [SP]/Phosphosphingolipids	835.691	835.66643	0.124	**[M+Na]$^+$**, [M+H]$^+$, [M+K]$^+$	$C_{47}H_{93}N_2O_6P$	2
SM 33:1;O2	Sphingolipids [SP]/Phosphosphingolipids	727.540	727.51511	0.164	**[M+K]$^+$**, [M+H]$^+$, [M+Na]$^+$	$C_{38}H_{77}N_2O_6P$	3
SM 36:1	Sphingolipids [SP]/Phosphosphingolipids	731.601	731.60611	0.236	**[M+H]$^+$**	$C_{41}H_{83}N_2O_6P$	2
SM 40:2;O2	Sphingolipids [SP]/Phosphosphingolipids	823.685	823.60921	0.392	**[M+K]$^+$**, [M+Na]$^+$, [M+H]$^+$	$C_{45}H_{89}N_2O_6P$	2
SM 40:0	Sphingolipids [SP]/Phosphosphingolipids	789.697	789.68421	0.576	**[M+H]$^+$**	$C_{45}H_{93}N_2O_6P$	2
SM 41:1;O2	Sphingolipids [SP]/Phosphosphingolipids	801.662	801.68459	0.646	**[M+H]$^+$**, [M+K]$^+$, [M+Na]$^+$	$C_{46}H_{93}N_2O_6P$	3
SM 41:2;O2	Sphingolipids [SP]/Phosphosphingolipids	837.707	837.62514	0.649	**[M+H]$^+$**, [M+K]$^+$, [M+Na]$^+$	$C_{46}H_{91}N_2O_6P$	3
SM 43:2;O2	Sphingolipids [SP]/Phosphosphingolipids	827.679	827.70003	0.675	**[M+H]$^+$**, [M+K]$^+$, [M+Na]$^+$	$C_{48}H_{95}N_2O_6P$	2
SM 43:2;O2	Sphingolipids [SP]/Phosphosphingolipids	879.702	879.67198	0.688	**[M+H]$^+$**, [M+K]$^+$, [M+Na]$^+$	$C_{49}H_{97}N_2O_6P$	2
SM 42:0;O2	Sphingolipids [SP]/Phosphosphingolipids	855.751	855.67221	0.697	**[M+H]$^+$**, [M+K]$^+$, [M+Na]$^+$	$C_{47}H_{97}N_2O_6P$	3
SM 44:1;O2	Sphingolipids [SP]/Phosphosphingolipids	881.662	881.68664	0.703	**[M+H]$^+$**, [M+K]$^+$, [M+Na]$^+$	$C_{49}H_{99}N_2O_6P$	2
SM 43:1;O2	Sphingolipids [SP]/Phosphosphingolipids	867.731	867.67247	0.919	**[M+H]$^+$**, [M+K]$^+$, [M+Na]$^+$	$C_{48}H_{97}N_2O_6P$	2
SM 42:1	Sphingolipids [SP]/Phosphosphingolipids	837.707	837.6823	0.931	**[M+Na]$^+$**, [M+H]$^+$, [M+K]$^+$	$C_{47}H_{95}N_2O_6P$	2
SM 36:0;O2	Sphingolipids [SP]/Phosphosphingolipids	755.531	755.60501	1.672	**[M+Na]$^+$**, [M+H]$^+$, [M+K]$^+$	$C_{41}H_{85}N_2O_6P$	2
SM 38:1;O2	Sphingolipids [SP]/Phosphosphingolipids	781.587	781.61959	0.255	**[M+Na]$^+$**, [M+H]$^+$, [M+K]$^+$	$C_{43}H_{87}N_2O_6P$	2
SM 41:2;O2	Sphingolipids [SP]/Phosphosphingolipids	821.661	821.65063	-0.462	**[M+Na]$^+$**	$C_{46}H_{91}N_2O_6P$	2
ST 23:4;O6	Sterol Lipids [ST]	553.332	553.29864	-4.282	**[M+H]$^+$**	$C_{29}H_{44}O_{10}$	3
CE 20:4	Sterol Lipids [ST]	673.444	673.58948	-3.325	**[M+H]$^+$**, [M+Na]$^+$	$C_{47}H_{76}O_2$	3
CE 22:5	Sterol Lipids [ST]	703.561	703.55057	-1.773	**[M+H]$^+$**, [M+Na]$^+$	$C_{43}H_{74}O_7$	3

ST 28:1;O;Hex	Sterol Lipids [ST]	879.771	879.70423	-0.946	**[M+Na]$^+$**, [M+K]$^+$	$C_{54}H_{96}O_7$	3
ST 24:2;O4	Sterol Lipids [ST]	429.283	429.24015	-0.064	**[M+K]$^+$**, [M+Na]$^+$, [M+H]$^+$	$C_{24}H_{38}O_4$	3
ST 30:1;O5	Sterol Lipids [ST]	493.325	491.37318	0.156	**[M+H]$^+$**	$C_{30}H_{50}O_5$	3
ST 24:0;O4	Sterol Lipids [ST]	395.316	395.31566	0.176	**[M+H]$^+$**	$C_{24}H_{42}O_4$	3
ST 24:1;O4;GlcA	Sterol Lipids [ST]	569.382	569.33238	0.333	**[M+H]$^+$**	$C_{30}H_{48}O_{10}$	3
ST 27:3;O	Sterol Lipids [ST]	383.373	383.33091	0.353	**[M+H]$^+$**	$C_{27}H_{42}O$	3
ST 30:1;O5	Sterol Lipids [ST]	493.401	493.38885	0.362	**[M+H]$^+$**	$C_{30}H_{52}O_5$	3
ST 28:0;O5	Sterol Lipids [ST]	467.265	467.20501	2.116	**[M+Na]$^+$**, [M+K]$^+$	$C_{25}H_{32}O_7$	3
Cholesterol sulfate	Sterol Lipids [ST]	465.305	465.30321	-2.568	**[M-H]$^-$**	$C_{27}H_{46}O_4S$	3
ST 21:1;O5	Sterol Lipids [ST]	479.292	479.28374	0.148	**[M-H]$^-$**	$C_{27}H_{44}O_5S$	3

Metabolites

**Compound**	**Biochemical class/Subclass**	**RapifleX® *m/z***	**SolariX® *m/z***	**Error (ppm)**	**Adduct (*)**	**Molecular Formula**	**MS Identification Level (**)**
Glutamine	Amino acids, peptides, and analogues	145.067	145.06197	0.856	**[M-H]$^-$**	$C_5H_{10}N_2O_3$	2
Glutamate	Amino acids, peptides, and analogues	146.068	146.04597	0.059	**[M-H]$^-$**, [M+Cl]$^-$	$C_5H_9NO_4$	2
Histidine	Amino acids, peptides, and analogues	154.047	154.06234	0.782	**[M-H]$^-$**	$C_6H_9N_3O_2$	2
Acetylcysteine	Amino acids, peptides, and analogues	162.023	162.02313	0.916	**[M-H]$^-$**	$C_5H_9NO_3S$	3
Methionine sulfoxide	Amino acids, peptides, and analogues	164.073	164.03881	0.398	**[M-H]$^-$**	$C_5H_{11}NO_3S$	3
Arginine	Amino acids, peptides, and analogues	173.025	173.10445	0.871	**[M-H]$^-$**	$C_6H_{14}N_4O_2$	2
Cysteinylglycine	Amino acids, peptides, and analogues	177.046	177.03404	0.501	**[M-H]$^-$**	$C_5H_{10}N_2O_3S$	2
Tyrosine	Amino acids, peptides, and analogues	180.051	180.06665	0.621	**[M-H]$^-$**, [M+Cl]$^-$	$C_9H_{11}NO_3$	2
Cystathionine	Amino acids, peptides, and analogues	221.083	221.06079	0.431	**[M-H]$^-$**	$C_7H_{14}N_2O_4S$	3
3-Nitrotyrosine	Amino acids, peptides, and analogues	225.059	225.05180	0.423	**[M-H]$^-$**	$C_9H_{10}N_2O_5$	3
Carnosine	Amino acids, peptides, and analogues	225.084	225.09952	0.567	**[M-H]$^-$**, [M+Cl]$^-$	$C_9H_{14}N_4O_3$	2
Gamma-Glutamylcysteine	Amino acids, peptides, and analogues	249.063	249.05532	0.907	**[M-H]$^-$**	$C_8H_{14}N_2O_5S$	3
Gamma-Glutamyl Glutamine	Amino acids, peptides, and analogues	274.121	274.10469	0.660	**[M-H]$^-$**	$C_{10}H_{17}N_3O_6$	2
Anserine	Amino acids, peptides, and analogues	239.061	239.11501	0.193	**[M-H]$^-$**	$C_{10}H_{16}N_4O_3$	2
Fructose 6-Phosphate	Carbohydrates and carbohydrate conjugates	259.031	259.02251	0.262	**[M-H]$^-$**	$C_6H_{13}O_9P$	2
Fructose 1,6-bisPhosphate	Carbohydrates and carbohydrate conjugates	339.003	338.98883	0.168	**[M-H]$^-$**	$C_6H_{14}O_{12}P_2$	2
N-Acetyl-L-aspartic acid	Carboxylic acids and derivatives	174.046	174.04087	0.962	**[M-H]$^-$**	$C_6H_9NO_5$	2
Citric acid	Carboxylic acids and derivatives	191.028	191.01986	0.456	**[M-H]$^-$**	$C_6H_8O_7$	2
Aconitic acid	Carboxylic acids and derivatives	173.011	173.00928	0.934	**[M-H]$^-$**, [M+Cl]$^-$	$C_6H_6O_6$	3
Pyroglutamic acid	Carboxylic acids and derivatives	128.042	128.03542	0.953	**[M-H]$^-$**	$C_5H_7NO_3$	2
Aspartate	Carboxylic acids and derivatives	132.033	132.03034	0.631	**[M-H]$^-$**, [M+Cl]$^-$	$C_4H_7NO_4$	2
2-Methylcitric acid	Carboxylic acids and derivatives	205.055	205.03546	0.976	**[M-H]$^-$**	$C_7H_{10}O_7$	3
Epsilon-(gamma-Glutamyl)-lysine	Carboxylic acids and derivatives	274.117	274.14096	1.222	**[M-H]$^-$**	$C_{11}H_{21}N_3O_5$	3
Glutathione	Carboxylic acids and derivatives	306.098	306.07664	0.686	**[M-H]$^-$**	$C_{10}H_{17}N_3O_6S$	2

Ascorbic acid	Dihydrofurans	175.025	211.00146	0.656	**[M+Cl]$^-$**, [M-H]$^-$	$C_6H_8O_6$	3
Galactonic acid	Hydroxy acids and derivatives	195.062	195.05133	0.329	**[M-H]$^-$**, [M+Cl]$^-$	$C_6H_{12}O_7$	3
Xanthine	Imidazopyrimidines	151.021	151.02627	0.683	**[M-H]$^-$**	$C_5H_4N_4O_2$	2
N-Acetylserotonin	Indoles and derivatives	217.082	217.09831	0.271	**[M-H]$^-$**	$C_{12}H_{14}N_2O_2$	2
Tryptophan	Indoles and derivatives	203.055	203.08251	-0.441	**[M-H]$^-$**	$C_{11}H_{12}N_2O_2$	2
Indoxyl sulfate	Organic sulfuric acids and derivatives	212.007	212.00241	0.509	**[M-H]$^-$**	$C_8H_7NO_4S$	2
N-Acetylneuraminic acid	Organooxygen compounds	308.098	308.09894	0.491	**[M-H]$^-$**, [M+Cl]$^-$	$C_{11}H_{19}NO_9$	2
Hydroxyphenylpyruvic acid	Phenylpyruvic acid derivatives	179.051	179.03536	0.211	**[M-H]$^-$**	$C_9H_8O_4$	3
p-Cresol sulfate	Phenylsulfates	187.021	187.00716	0.453	**[M-H]$^-$**	$C_7H_8O_4S$	3
Inosine	Purine nucleosides	267.091	267.07366	0.957	**[M-H]$^-$**	$C_{10}H_{12}N_4O_5$	2
Xanthosine	Purine nucleosides	283.094	283.06854	1.154	**[M-H]$^-$**	$C_{10}H_{12}N_4O_6$	2
Guanosine	Purine nucleosides	282.069	282.08451	0.285	**[M-H]$^-$**	$C_{10}H_{13}N_5O_5$	3
Adenosine Triphosphate (ATP)	Purine nucleotides	506.007	505.98910	1.241	**[M-H]$^-$**	$C_{10}H_{16}N_5O_{13}P_3$	2
Cyclic AMP (cAMP)	Purine ribonucleotides	328.077	328.04567	0.629	**[M-H]$^-$**	$C_{10}H_{12}N_5O_6P$	3
Adenosine monophosphate (AMP)	Purine ribonucleotides	346.061	346.05608	0.688	**[M-H]$^-$**	$C_{10}H_{14}N_5O_7P$	2
Inosinic acid	Purine ribonucleotides	347.055	347.04013	-4.801	**[M-H]$^-$**	$C_{10}H_{13}N_4O_8P$	3
Uric acid	Purines and purine derivatives	167.024	167.02118	0.984	**[M-H]$^-$**	$C_5H_4N_4O_3$	2
Hypoxanthine	Purines and purine derivatives	135.043	135.03132	-0.302	**[M-H]$^-$**	$C_5H_4N_4O$	2
Niacinamide	Pyridine nucleotides	121.040	121.04016	0.126	**[M-H]$^-$**	$C_6H_6N_2O$	2
Deoxycytidine	Pyrimidine nucleosides	226.084	226.08354	1.075	**[M-H]$^-$**, [M+Cl]$^-$	$C_9H_{13}N_3O_4$	3
Deoxyuridine	Pyrimidine nucleosides	227.059	227.06752	1.043	**[M-H]$^-$**	$C_9H_{12}N_2O_5$	3
Cytidine	Pyrimidine nucleosides	242.062	242.07819	0.865	**[M-H]$^-$**	$C_9H_{13}N_3O_5$	2
Uridine	Pyrimidine nucleosides	243.062	243.06248	1.001	**[M-H]$^-$**, [M+Cl]$^-$	$C_9H_{12}N_2O_6$	2
5-Carboxy-2'-deoxyuridine	Pyrimidine nucleosides	271.059	271.05742	0.377	**[M-H]$^-$**	$C_{10}H_{12}N_2O_7$	2
Guanosine monophosphate (GMP)	Pyrimidine nucleosides	362.057	362.05092	0.747	**[M-H]$^-$**	$C_{10}H_{14}N_5O_8P$	2
5-Methylcytidine	Pyrimidine nucleosides	256.064	256.09419	0.756	**[M-H]$^-$**	$C_{10}H_{15}N_3O_5$	3
Uridine 5'-monophosphate (UMP)	Pyrimidine nucleotides	323.051	323.02887	0.526	**[M-H]$^-$**	$C_9H_{13}N_2O_9P$	2
1-hydroxytacrine	Quinolines and derivatives	213.114	213.10342	0.389	**[M-H]$^-$**	$C_{13}H_{14}N_2O$	2
Adenosine diphosphate (ADP)	Purine ribonucleotides	426.043	426.02224	0.237	**[M-H]$^-$**	$C_{10}H_{14}N_5O_7P$	2

(*) Adduct relating to the *m/z* columns is reported in bold**; (**)** Metabolites were putatively annotated with MSI level 2 of the Metabolomics Standards Initiative recommendations [25] with accurate mass/spectral similarity/isotopic fine structure. Where MS/MS data were not available, they were tentatively assigned to chemical classes and reported with MSI level 3.

Table S2. Additional RP-UHPLC-TIMS-MS to enforce the annotation of the statistical relevant lipids derived from PLS-DA and/or spatial segmentation analysis. Lipids were reported with long name nomenclature if MS/MS spectra contained information about fatty acyl composition, otherwise a short name nomenclature was adopted.

**Short name**	**Long name**	**m/z**	**Error (ppm)**	**Adduct (*)**	**CCS (Å²)**	**Molecular Formula**
Cer 42:2;O	Cer 18:1;O/24:1	632.629	-1.046	**[M+H]$^+$**, [M+Na]$^+$	281.5	$C_{42}H_{81}NO_2$
DG O-36:4	DG O-36:4	603.534	-1.032	**[M+H]$^+$**, [M+Na]$^+$	261.5	$C_{39}H_{70}O_4$
LPC 16:0	LPC 16:0-SN1	496.339	-0.785	**[M+H]$^+$**, [M+Na]$^+$, [M+K]$^+$	232.7	$C_{24}H_{50}NO_7P$
PC 30:0	PC 15:0_15:0	744.495	0.671	**[M+K]$^+$**, [M+Na]$^+$, [M+H]$^+$	281.8	$C_{38}H_{76}NO_8P$
PC 32:0	PC 16:0_16:0	772.526	1.293	**[M+K]$^+$**, M+Na]$^+$, [M+H]$^+$	287.1	$C_{40}H_{80}NO_8P$
PC 32:1	PC 16:0_16:1	770.511	1.297	**[M+K]$^+$**, [M+Na]$^+$, [M+K]$^+$	284.2	$C_{40}H_{78}NO_8P$
PC 34:1	PC 16:0_18:1	798.542	1.252	**[M+K]$^+$**, [M+Na]$^+$, [M+H]$^+$	290.3	$C_{42}H_{82}NO_8P$
PC 34:3	PC 16:0_18:3 or PC 16:1_18:2	756.554	0.885	**[M+H]$^+$**	283.4/ 284	$C_{42}H_{78}NO_8P$
PC 32:2 or PE 35:2	PC 16:1_16:1 or PE 9:0_26:2	730.540	1.846	**[M+H]$^+$**	280.7/277.8	$C_{40}H_{76}NO_8P$
PC 33:2 or PE 36:2	PC 17:0_16:2 or PE 18:0_18:2 or PE 18:1_18:1	744.554	0.623	**[M+H]$^+$**, [M+Na]$^+$	283.6/281.6/281	$C_{41}H_{78}NO_8P$
PC 34:2	PC 17:1_17:1	758.563	1.318	**[M+K]$^+$**, [M+Na]$^+$, [M+H]$^+$	286.8	$C_{42}H_{80}NO_8P$
PC 38:3	PC 18:0_20:3 or PC 20:1_18:2	812.618	1.761	**[M+H]$^+$**, [M+K]$^+$, [M+Na]$^+$,	297.3/ 296.9	$C_{46}H_{86}NO_8P$
PC 21:1	-	578.412	0.064	**[M+H]$^+$**, [M+Na]$^+$, [M+K]$^+$	-	$C_{29}H_{56}NO_8P$
PC 25:1;O	-	650.459	0.183	**[M+H]$^+$**, [M+Na]$^+$, [M+K]$^+$	-	$C_{33}H_{64}NO_9P$
PC O-32:2	-	551.511	0.007	**[M+H]$^+$**,[M+Na]$^+$	-	$C_{35}H_{66}O_4$
PC O-42:6	-	870.655	0.292	**[M+Na]$^+$**, [M+K]$^+$, [M+H]$^+$	-	$C_{50}H_{90}NO_7P$
PC O-44:5	PC O-44:5	900.683	1.109	**[M+Na]$^+$**, [M+K]$^+$	312.6	$C_{52}H_{96}NO_7P$
PE 34:0	PE 18:0_16:0	718.554	0.799	**[M-H]$^-$**	285.1	$C_{39}H_{78}NO_8P$
PE 36:3	PE 12:0_24:3 or PE 18:1_18:2	742.539	1.289	**[M+H]$^+$**	279.2/ 277.6	$C_{41}H_{76}NO_8P$
PE 40:2	PE 18:1_22:1	800.617	1.249	**[M+H]$^+$**	295.1	$C_{45}H_{86}NO_8P$
PE 42:7	-	818.479	-2.834	**[M+H]$^+$**	-	$C_{47}H_{80}NO_8P$
PE O-38:6	PE O-16:1_22:5	750.544	0.619	**[M+H]$^+$**, [M+Na]$^+$	279.3	$C_{43}H_{76}NO_7P$
PS O-30:2	-	712.461	0.132	**[M+Na]$^+$**, [M+K]$^+$, [M+H]$^+$	-	$C_{36}H_{68}NO_9P$
PS 36:1	-	524.334	1.102	**[M-H]$^-$**	-	$C_{24}H_{48}NO_9P$
SM 44:1;O2	SM 18:1;2O/26:0	881.687	0.589	**[M+K]$^+$**, [M+Na]$^+$, [M+H]$^+$	313.8	$C_{49}H_{99}N_2O_6P$
SM 41:2;O2	SM 18:2;2O/23:0	821.651	0.453	**[M+Na]$^+$**	303.4	$C_{46}H_{91}N_2O_6P$
SM 43:2;O2	SM 18:2;2O/25:0	827.693	0.806	**[M+H]$^+$**, [M+K]$^+$, [M+Na]$^+$	309.4	$C_{48}H_{95}N_2O_6P$
SM 36:1;O2	SM 18:1;2O/18:0	731.598	-0.135	**[M+H]$^+$**	292.5	$C_{41}H_{83}N_2O_6P$
SM 38:1;O2	SM 18:1;2O/20:0	781.618	0.022	**[M+Na]$^+$,** [M+K]$^+$, [M+H]$^+$	297.8	$C_{43}H_{87}N_2O_6P$
SM 34:2;O2	SM 16:2;2O/18:1	723.541	-0.312	**[M+Na]$^+$**, [M+H]$^+$, [M+K]$^+$	283	$C_{39}H_{77}N_2O_6P$
SM 38:0;O2	-	761.595	-0.477	**[M+H]$^+$**	-	$C_{43}H_{89}N_2O_6P$
TG 54:4	TG 18:1_18:1_18:2	883.775	0.785	**[M+H]$^+$**	324.6	$C_{57}H_{102}O_6$
TG 53:2	TG 17:0_18:1_18:1	895.774	0.116	**[M+Na]$^+$,** [M+K]$^+$	324.9	$C_{56}H_{104}O_6$
TG 52:2	TG 16:0_18:1_18:1	881.757	0.259	**[M+Na]$^+$,** [M+K]$^+$	322.5	$C_{55}H_{102}O_6$

TG 54:3	TG 18:1_18:1_18:1	907.773	0.561	**[M+Na]$^+$,** [M+K]$^+$, [M+H]$^+$	326.8	$C_{57}H_{104}O_6$
TG 56:5	TG 18:1_18:1_20:3	909.792	1.163	**[M+H]$^+$**, [M+Na]$^+$, [M+K]$^+$	329.4	$C_{59}H_{104}O_6$
TG 58:9	TG 18:1_18:2_22:6	929.759	0.384	**[M+H]$^+$**, [M+Na]$^+$, [M+K]$^+$	328.6	$C_{61}H_{100}O_6$

(*) Adduct relating to the *m/z* columns is reported in bold.

Table S3. Additional HILIC-HRMS/MS to enforce the annotation of the statistical relevant non-polar and polar metabolites derived from PLS-DA and/or spatial segmentation analysis.

**Compound name**	***m/z***	**Error (ppm)**	**Adduct (*)**	**MS/MS**	**Molecular Formula**
CAR 16:0	400.341	0.209	**[M+H]$^+$**	341.2693, 85.0286, 60.0815	$C_{23}H_{45}NO_4$
LPC 15:0	480.309	0.119	**[M-H]$^-$**	256.2335, 242.0789, 167.9525	$C_{23}H_{48}NO_7P$
FA 18:2	279.233	0.429	**[M-H]$^-$**	261.2238, 96.9598	$C_{18}H_{32}O_2$
FA 20:4	303.233	0.519	**[M-H]$^-$**	259.2423, 231.2108, 285.2182	$C_{20}H_{32}O_2$
FA 22:6	327.233	0.859	**[M-H]$^-$**	283.2436, 229.1972, 59.0128	$C_{22}H_{32}O_2$
FA 18:0	283.263	0.519	**[M-H]$^-$**	201.2264	$C_{18}H_{36}O_2$
Glutamine	145.061	0.479	**[M-H]$^-$**	127.0497,	$C_5H_{10}N_2O_3$
Glutamate	146.045	0.169	**[M-H]$^-$**	128.0345, 102.0545	$C_5H_9NO_4$
Adenosine diphosphate (ADP)	426.021	0.709	**[M-H]$^-$**	346.0567, 134.0464, 96.968	$C_{10}H_{15}N_5O_{10}P_2$
Adenosine monophosphate (AMP)	346.055	0.129	**[M-H]$^-$**	134.047, 96.968, 79.9578	$C_{10}H_{14}N_5O_7P$
Uridine monophosphate (UMP)	323.027	1.089	**[M-H]$^-$**	211.0011, 111.0208, 96.968, 79.9578	$C_9H1_3N_2O_9P$
Guanosine monophosphate (GMP)	362.050	0.747	**[M-H]$^-$**	150.0412, 108.0201 96.968	$C_{10}H_{14}N_5O_8P$
Xanthine	151.025	0.749	**[M-H]$^-$**	108.0189, 126.0269	$C_5H_4N_4O_2$
Hypoxanthine	135.030	1.129	**[M-H]$^-$**	117.0318, 92.0239, 65.0139	$C_5H_4N_4O$
Cytidine	242.077	0.379	**[M-H]$^-$**	110.0359	$C_9H_{13}N_3O_5$
Aspartate	132.029	1.219	**[M-H]$^-$**	115.0024, 88.04, 74.0125	$C_4H_7NO_4$

(*) Adduct relating to the *m/z* columns is reported in bold.
